# Nano‐Oil‐Barrier‐Based Fluttering Triboelectric Nanogenerator

**DOI:** 10.1002/advs.202502278

**Published:** 2025-05-20

**Authors:** Deokjae Heo, Jiwoong Hur, Hyeonho Cho, Kyunghwan Cha, Jaeung Choi, Moonhyun Choi, Jinkee Hong, Sunghan Kim, Sangmin Lee

**Affiliations:** ^1^ Center for Systems Biology Massachusetts General Hospital, Harvard Medical School Boston MA 02114 USA; ^2^ School of Mechanical Engineering Chung‐Ang University 84, Heukseok‐ro, Dongjak‐gu Seoul 06974 Republic of Korea; ^3^ Department of Chemical & Biomolecular Engineering College of Engineering Yonsei University 50 Yonsei‐ro, Seodaemun‐gu Seoul 03722 South Korea

**Keywords:** durability, energy harvesting, lubrication, nano‐oil‐barrier, stability, triboelectric nanogenerator

## Abstract

In the field of triboelectric nanogenerators (TENGs), the application of a thin lubricant layer on the contact surface and its maintenance for long‐term cycling remain important challenges for improving the mechanical‐electrical stability of TENGs. Herein, a simple and innovative approach is proposed to solve this dilemma using commercial oil‐absorbing sheets and oil infusion steps. In particular, a wind‐driven nano‐oil‐barrier‐based fluttering triboelectric nanogenerator (NF‐TENG) is developed. The nano‐oil barrier (of nanoscale thickness) of NF‐TENG is thoroughly analyzed using atomic force microscopy imaging and electrical‐mechanical measurement/calculation results. Compared with other control groups, only NF‐TENG maintains 95% output performance from 100% initial output performance, and device damage is minimized even after 970,000 cycles. The mechanism of NF‐TENG and its differences from previous studies are established. NF‐TENG is optimized and studied for various design variables and wind speeds. NF‐TENG generated a peak power of 468 µW with 100 Hz and an average power of 166 µW at optimum load resistance, under a breeze wind speed of 6 m s^−1^. NF‐TENG demonstrates its applications in two real‐life scenarios: 1) wind harvesting at a rooftop vent pipe for outdoor temperature‐humidity sensing, and 2) wind harvesting during bicycle riding for safety light illumination.

## Graphical abstract

This work proposes a wind‐driven nano‐oil‐barrier‐based fluttering triboelectric nanogenerator (NF‐TENG). A nano‐oil barrier is a simple and innovative strategy to maintain a thin lubricant layer for long‐term cycling based on commercial oil‐absorbing sheets and oil‐infusion steps, which can improve both mechanical and electrical stability of TENGs.

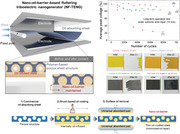

## 1 Introduction

Triboelectric nanogenerators (TENGs) are energy harvesting technologies based on the principles of contact electrification between two different surfaces (e.g., solid–solid, solid–liquid) and electrostatic induction. TENGs convert various ambient mechanical energy sources (wind, human motion, vibration, water droplet, etc.) into available electrical energy.^[^ ^1^ ^]^ In particular, TENGs have frequently utilized wind energy as the most abundant and renewable energy source. Wind‐driven TENGs are an attractive solution in the low wind‐speed range because TENGs inherently have high efficiency under small inputs compared to other principle‐based nanogenerators (e.g., electromagnetic, piezoelectric).^[^ ^2^ ^]^ Wind‐driven TENGs are classified based on blade/ball rotation^[^ ^3^ ^]^ and sheet fluttering.^[^ ^4^ ^]^ Among them, the sheet fluttering type has been widely studied recently because it generates a high‐Hz peak output through the rapid vibration of an extremely light and thin sheet, even given a breeze wind input.^[^ ^5^ ^]^ The energy harvested by TENGs is used as a small‐scale, independent, and sustainable power supply for distributed commercial low‐power electronics and sensors.

However, TENGs have fundamental limitations, such as friction and wear. This results in poor mechanical‐electrical durability and stability of TENG. In this regard, researchers have explored innovative approaches such as designing noncontact TENG, introducing robust triboelectric surface materials/structures, and adding oil as a surface lubricant.^[^ ^6^ ^]^ Compared with the first and second methods, the last method is most commonly used for TENG because it can easily and intuitively reduce friction and wear in daily life. However, it is difficult to control the thickness of the oil coating; thus, excessive oil is applied to the surface of the TENG, leaving residual oil that hinders its electrical output.^[^ ^7^ ^]^ To this end, a breakthrough is needed to develop TENG by maintaining a thin oil lubrication layer that can improve both mechanical and electrical performance.

In this study, a wind‐driven nano‐oil‐barrier‐based fluttering triboelectric nanogenerator (NF‐TENG) with long‐term mechanical‐electrical stability was developed. NF‐TENG consists of a commercial oil‐absorbing sheet, top and bottom electrodes, a fixed flagpole, and a substrate. The commercial oil‐absorbing sheet has a polymer‐based porous structure and a thin oil‐coated state (called the nano‐oil‐layer in this study) via the proposed simple oil‐infusion steps. As a result, NF‐TENG consistently maintains the nano‐oil barrier even after long‐term fluttering motion, leading to minimized device breakage and outstanding output stability. The surface structures with and without oil were carefully investigated using atomic force microscopy (AFM), and the thickness of the oil was determined based on mechanical measurements and calculations. The electricity‐generation mechanism of NF‐TENG was proposed based on the high‐speed mechanical motion of the sheet. The design variables of NF‐TENG were thoroughly analyzed and optimized based on electrical output measurements. Finally, the peak output and charged energy of NF‐TENG demonstrated its functionality as a power source in daily life for commercial temperature/humidity sensors and LED array‐based safety lights in two wind scenarios.

## 2 Results and Discussion

### 2.1 Main Concept of NF‐TENG

**Figure 1:**
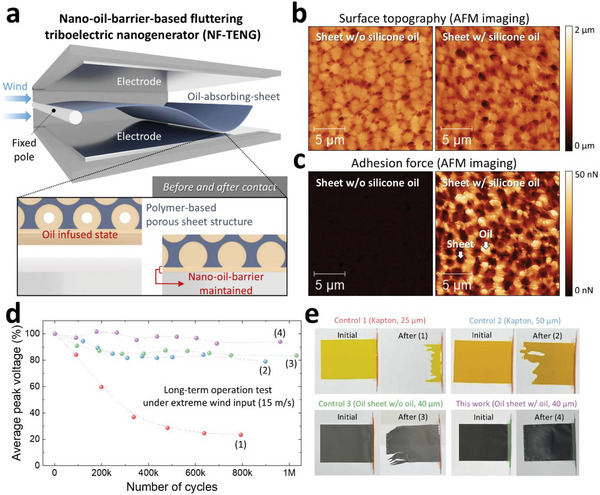
Nano‐oil‐barrier‐based fluttering triboelectric nanogenerator (NF‐TENG). a) 3D structure of NF‐TENG b) Surface topography (z‐scale: 2 µm), and c) adhesive force (z‐scale: 50 nN) between the oil‐absorbing sheet with and without silicone oil based on atomic force microscope (AFM) with scale bars of 5 µm. d) Electrical stability and e) mechanical durability test results of this work and the three control groups, for long‐term working cycles.

NF‐TENG is composed of a commercial oil‐absorbing sheet (polypropylene/polyethylene mixed polymer, internal porous structure) and a flagpole connected to the end of the sheet, top electrode (aluminum), bottom electrode (aluminum), and substrate (acrylic) (**Figure** **1**a). When wind is applied to NF‐TENG, the flagpole remains fixed and the sheet oscillates rapidly. Notably, in this study, the sheet surface maintained the nano‐oil barrier (thin oil‐coated layer with nanoscale thickness) even at the moment of contact motion. This nano‐oil barrier provided a sustainable lubrication effect and electric output generation stability of NF‐TENG compared with conventional methods.

Figure 1b–c contrasts surface topography and adhesion force between the oil‐absorbing sheet with and without silicone oil (one of the representative oil materials for lubrication), based on atomic force microscope (AFM). Analyzing how oil is contained in an oil‐absorbing sheet is generally challenging using typical microscopic methods because oil distorts the surface structure in the resulting images. Therefore, in this study, the pinpoint mode AFM was applied to investigate the topography and adhesive force of the sheet in both cases with and without silicone oil.

The surface topography of the sheet without silicone oil was clearly scanned using a pinpoint‐mode AFM. However, the surface topography of the sheet with silicone oil appeared indistinct compared with that of the sheet without silicone oil. The comparison of AFM topography images with and without silicone oil confirmed the presence of silicone oil on the surface of the sheet. Adhesive force measurements were conducted to further analyze the morphology of the oil‐absorbing sheet. For the sheet without silicone oil, the adhesive force was nearly zero because of the weak interaction between the AFM tip and the sheet surface. In contrast, for the sheet with silicone oil, the adhesive force varied significantly between the regions with silicone oil and the sheet itself. The difference in adhesive force between the sheets with and without silicone oil is clearly demonstrated in the adhesive force distribution graph of the AFM image (Figure S1, Supporting Information). By comparing the topographic images with the adhesive force images, it was evident that the silicone oil was effectively absorbed into the porous structure of the sheet. This absorption is expected to provide a sustainable lubricating effect during NF‐TENG operation.

Figure 1d,e shows the differences in electrical stability and mechanical durability for long‐term working cycles between this implementation and the three control groups. In this test, a wind input of 15 m s^−1^ was used, assuming extreme wind conditions. This study used a 40 µm commercial oil‐absorbing‐sheet w/oil, and control groups 1–3 utilized a 25 µm Kapton sheet, a 50 µm Kapton sheet, and the commercial oil‐absorbing sheet identical to that used in this study but w/o oil, respectively. As a result, the average peak voltage in this work maintained a 95% level, even after 970 000 cycles from 100% initial output (Figure 1d, (4)). In contrast, the average peak voltage of control 1 drastically decreased from 100% initial output to 23% after 800 000 cycles (Figure 1d, (1)). Controls 2–3 showed similar decreases from 100% initial output to 78% and 82% output after 900 000 and 1060 000 cycles, respectively (Figure 1d, (2–3)). The raw voltage graphs for Figure 1d are presented in Figure S2 (Supporting Information). This output stability is closely related to mechanical durability. As shown in Figure 1e, Control 1 was completely destroyed after 800 000 cycles. In contrast, this implementation maintained its original shape without significant physical damage, even after 970 000 cycles. Controls 2 and 3 exhibited slight mechanical fractures after long‐term working cycles. The data in Figure 1d,e demonstrates the electrical and mechanical stability of NF‐TENG owing to its unique nano‐oil barrier.

The environmental impact of long‐term operation is an important aspect. If oil continues to leak or the Al electrodes are severely worn and produce Al wear particles/debris after the long‐term operating cycle, it may affect the surrounding ecosystems. In this regard, first, the oil thickness and weight of the oil‐absorbing sheet were measured before and after the long‐term operating cycle. As a result, the oil thickness was maintained from 100% (initial) to 99.3% (after 1000 000 cycles), and the oil‐absorbing sheet weight was maintained from 6.81 g (initial) to 6.81 g (after 1000 000 cycles) (Figure S3a, Supporting Information). These experimental results demonstrate that there is no oil leakage from the sheet to the surroundings or loss of the sheet itself. Each of the oil thickness and sheet weight was measured using AFM and an analytical balance (AS220 R2 plus, RADWAG Co., Poland). Next, the Al electrode surface was observed using an optical microscope (OLYMPUS Co., USA), and an Al wear particle/debris absorption test was conducted for the long‐term operating cycle. As a result, even after 1000 000 cycles, there was no significant wear in the optical microscope image (Scale bar: 200 µm) (Figure S3b, Supporting Information). In addition, an adhesive sheet to which Al wear particles/debris can easily stick was installed right behind the NF‐TENG, and even after 1000 000 cycles, there were no Al wear particles/debris (Figure S3c, Supporting Information) on the adhesive sheet. These results collectively confirm that the environmental impact of long‐term operation is negligible. There was no detectable oil leakage and sheet loss, or Al wear particle/debris generation even after 1000 000 cycles, indicating the robustness and environmental stability of the device during prolonged use. Nevertheless, if any issues occur during long‐term operation, the biodegradability of the materials themselves becomes an important factor influencing environmental impact. In this study, olive oil (among the tested lubricant materials) and green tea/wood pulp/hanji (among the oil‐absorbing sheet materials) are biodegradable, i.e., more environmentally friendly choices. However, the three electrode materials tested (aluminum, nickel, and copper) were selected for their high conductivity and electrostatic induction, and are not biodegradable. Therefore, research to further improve the overall biodegradability, including electrode materials, can be a separate future study in terms of emphasizing environmental friendliness.

Table S1 (Supporting Information) presents a quantitative comparison between existing lubrication TENGs and NF‐TENG (this work). As a result, only NF‐TENG showed the nanometer‐level lubrication thickness, which was quantitatively measured and analyzed by AFM. Furthermore, NF‐TENG showed the least output performance decay even after the highest number of test operating cycles, 1000 000 cycles.

### 2.2 Mechanism of NF‐TENG

**Figure 2:**
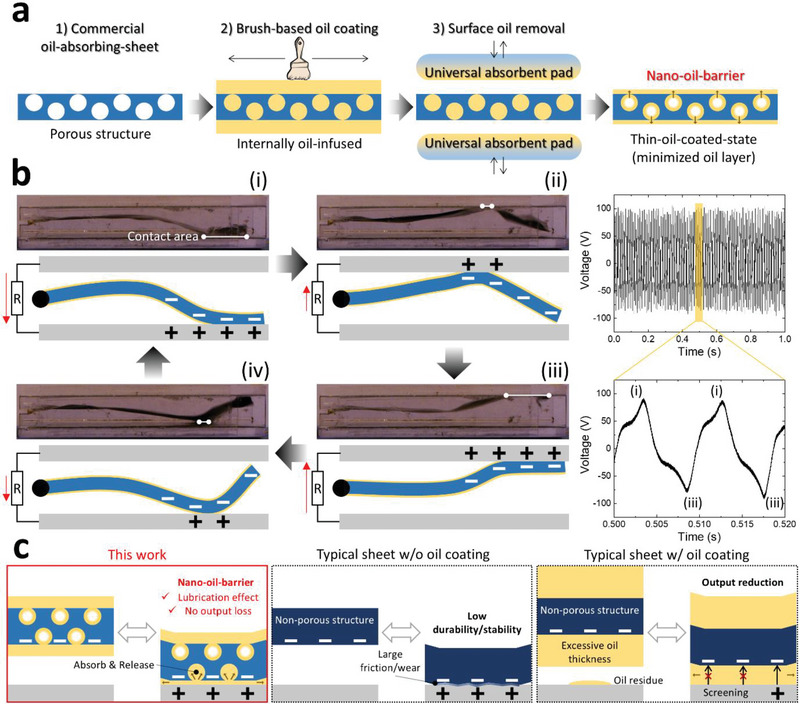
Mechanism and novelty of NF‐TENG. a) Oil‐infusion step to form nano‐oil‐barrier. b) Electrical mechanism of NF‐TENG according to the high‐speed fluttering motion of the sheet. c) The main differences in the mechanical/electrical mechanism between this study and other previous TENG studies.

NF‐TENG is practical because it uses commercial oil‐absorbing sheets and requires simple oil injection steps (**Figure** **2**a). First, a commercial oil‐absorbing sheet with a microporous structure received a brush‐based oil coating for oil infusion. However, this results in an excessively thick oil layer on the outer surface of the sheet. To address this, a universal oleophilic absorbent pad with a large oil‐absorbing capacity is used to completely remove surface oil by pressing and releasing vertically several times. At this time, some of the oil filling the pores escapes to the surface, naturally forming a nano‐oil barrier. This nano‐oil barrier is a thin oil‐coated state minimized to nanoscale thickness that persists even when repeated mechanical contact friction occurs.

NF‐TENG generates electricity according to the fluttering motion of the sheet (Figure 2b). When the wind above a critical flow rate level passes through the inside of NF‐TENG, the sheet oscillates rapidly, which is caused by the flow‐induced pressure difference on the sheet surface, repeated impact‐rebound with the top and bottom surfaces of the channel. The mechanical behavior of the sheet was analyzed using a high‐speed camera and classified into four types of contact‐separation motions. In the initial state, it is assumed that the sheet and electrode are negatively and positively charged, respectively, owing to triboelectrification by pre‐operation, and the charge of the oil itself is neglected. The triboelectric effect is based on the electron affinity difference between the PP/PE mixed polymer and Al.^[^ ^8^ ^]^ This can also be explained using the electron‐cloud‐potential‐well model.^[^ ^9^ ^]^ The atoms of the PP/PE mixed polymer and Al have their own potential wells in which electrons are weakly bound to form each electron cloud. Before the PP/PE‐mixed polymer and Al contact, the electrons cannot escape from their respective potential wells because of the trapping effect. However, if the PP/PE‐mixed polymer and Al contact each other, their electron clouds overlap and their respective potential wells transform into an asymmetric double‐potential well, allowing electron transfer from Al to the PP/PE‐mixed polymer. In the first motion (Figure 2b–i), as the negatively charged sheet contacts the bottom electrode with a maximized contact area and positive charges are induced toward the bottom electrode, electrical current flows from the top electrode to the bottom electrode. In the second motion, the sheet is completely separated from the bottom electrode and contacts the top electrode (Figure 2b‐ii), whereas in the third motion, it makes maximum contact with the top electrode (Figure 2b‐iii). At this time, current flows from the bottom electrode to the top electrode. In the fourth motion, the sheet moves away from the top electrode and comes into contact with the bottom electrode (Figure 2b‐iv). As it returns to the first motion, the contact area with the bottom electrode becomes maximum (Figure 2b‐i), and then the current flows from the top electrode to the bottom electrode again, owing to electrostatic induction. This working cycle produces an alternating current (AC) peak output waveform. As a result, NF‐TENG generates a high‐voltage and high‐frequency AC peak output by the flutter motion (100 V and 100 Hz at 6 m s^−1^). Details of the high‐speed motion are shown in Movie S1 (Supporting Information).

The key differences in the mechanical‐electrical mechanism between this work and previous TENG studies are illustrated in Figure 2c. In the case of existing TENG, where oil is not used on the triboelectric surface, friction and wear are high, which reduces the durability and stability of TENG. To solve these problems, many TENGs have applied oils to the triboelectric surfaces. However, owing to the non‐porous structure of the triboelectric surface, there are other sub‐problems, such as excessive oil surface thickness, oil residue, and resulting instability of the oil layer. Therefore, the electrical output of TENGs is reduced by the screening effect of the oil layer.

**Figure 3:**
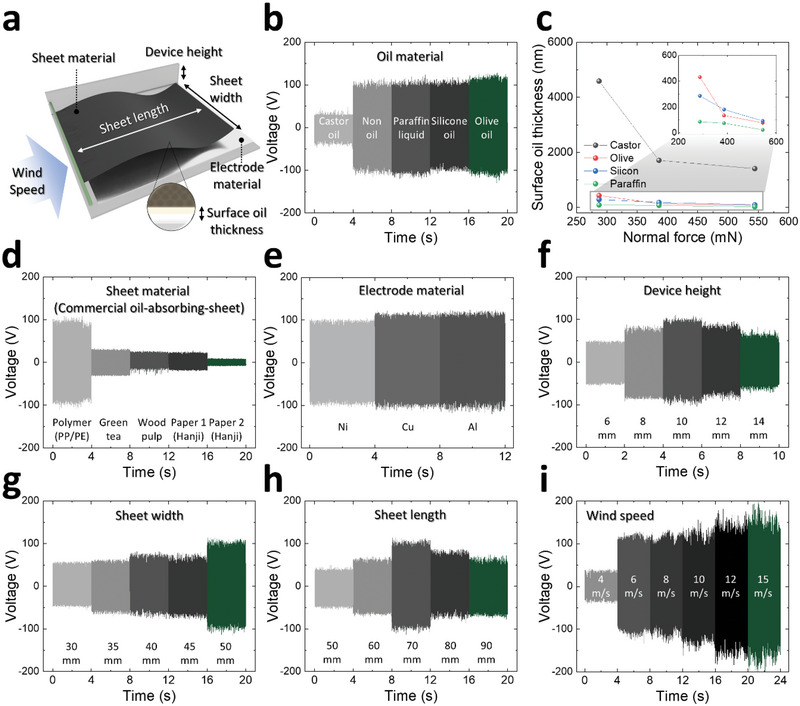
Design variables of NF‐TENG. a) 3D illustration for each design variable (oil material, sheet material, electrode material, device height, sheet width, sheet length, and wind speed) of NF‐TENG. b) Output voltage and c) surface oil thickness for different oil materials. Output voltage graph for various d) sheet material, e) electrode material, f) device height, g) sheet width, h) sheet length, and i) wind speed conditions.

Additional experiments (Figures S4 and S5, Supporting Information) showed that a typical nonporous sheet (e.g., Kapton) was unsuitable for the oil‐infusion steps shown in Figure 2a. Specifically, after brush‐based oil coating, the oil was completely removed, and the lubricating effect was immediately lost if the surface oil was absorbed by the universal absorbent pad several times. When only the brush‐based oil coating was applied, the oil layer remained thick and yielded a residue that reduced the electrical output by half. In contrast, the commercial oil‐absorbing sheet (this study) maintained the nano‐oil‐barrier layer naturally formed by the internal pore structure even after the surface oil was completely removed using the universal absorbent pad; thus, the electrical output did not decrease. The detailed measurement results for this nano‐oil barrier layer are shown in **Figure** **3**c.

### 2.3 Design Variables of NF‐TENG

NF‐TENG has several important design variables (oil material, sheet material, electrode material, device height, sheet width, and sheet length) that need to be optimized (Figure 3a). First, the output voltages of the non‐oil (bare sheet) and various oil materials (silicone oil, olive oil, paraffin liquid, and castor oil) were measured (Figure 3b). In the case without oil, the average peak voltage was measured as 95.8 V. When silicone oil, olive oil, and paraffin liquid were applied to the sheet, NF‐TENG generated average peak voltages of 97.4, 111, and 97.3 V, respectively. It is noteworthy that NF‐TENG w/silicone, olive, and paraffin liquid oils exhibited a high output similar to that of the non‐oil sheet, despite the presence of an oil layer. However, when castor oil was applied to the sheet, the average peak voltage decreased to 26.6 V. This drastic reduction in the output of with castor oil was related to the surface oil thickness of the sheet.

In this regard, the oil thickness of the sheet surface was measured to demonstrate its lubricant effects while maintaining TENG performance. This was achieved by using a pin‐on‐disk test.^[^ ^10^ ^]^ Sheets impregnated with different oils (castor, olive, silicone, or paraffin) were secured to the rotating part of the substrate holder. An Al tip, identical to the material used in NF‐TENG, was employed. Normal forces of 287, 386, and 544 mN were applied to simulate low contact pressure between the Al electrode and the oil‐absorbing sheet during actual NF‐TENG operation. During the pin‐on‐disk test, the surface oil thickness (*h*) was calculated using the following equation:^[^ ^11^ ^]^

(1) $$h=\frac{\rho \eta Av}{\mu_{c}N}\;$$

where *ρ* is the density, *η* is the kinematic viscosity, *µ_c_* is the coefficient of friction (CoF), *N* is the normal load, *ν* is the sliding velocity, and *A* is the contact area between the pin and the sheet. Kinematic viscosity (*η*) was determined using a rotational viscometer (Figure S6a, Supporting Information), while CoF, normal load, and sliding velocity were monitored during the pin‐on‐disk test using a tribometer (Figure S6b, Supporting Information). The contact area was calculated using the Hertz contact model:^[^ ^12^ ^]^

(2) $$a={\left(\frac{3NR}{4E^{*}}\right)}^{1/3}\;$$

where *a* is the contact radius, *R* is the pin radius (3 mm as provided by the manufacturer), and E^*^ is the effective Young's modulus. E^*^ was derived from Equation (3):

(3) $$\frac{1}{E^{*}}=\frac{1-\upsilon_{1}^{2}}{E_{1}}+\frac{1-\upsilon_{2}^{2}}{E_{2}}$$

where *E_1_* and *E_2_* represent the Young's moduli of the sheet and pin, respectively (*E_1_* was measured as 72 MPa and *E_2_* assumed as 76 GPa), and *ν_1_*, *ν_2_* are their Poisson ratios (both assumed as 0.3).

The surface oil thicknesses were compared for the sheets impregnated with various oils (Figure 3c). The sheets with castor oil exhibited the highest surface oil thickness because of their higher viscosity compared to the other oils. High viscosity hinders the absorption and release of oil within the porous structure of the sheet, resulting in greater surface oil thickness. However, greater oil thickness adversely affected TENG performance (Figure 3b).

Atomic force microscopy was employed to further evaluate the surface oil thickness in the absence of direct electrode contact.^[^ ^12^, ^13^ ^]^ A force‐distance (FD) curve test was conducted on the oil‐absorbing sheets (Figures S7a and S8, Supporting Information). During the FD test, the first snap‐in phenomenon occurred at the oil surface, whereas the second snap‐in occurred at the sheet surface, owing to the attractive forces between the tip and surface. The surface oil thickness (*t*) was calculated from the difference in the AFM z‐scanner distance (*Δz*) and cantilever bending deflection (*Δω*) between the two snap‐in phenomenon:

(4) t=Δz+Δω

The bending deflection was derived using:^[^ ^14^ ^]^

(5) $$\omega =\frac{F}{k}\;$$

where *F* is the applied force, and *k* is the spring constant of the AFM cantilever. Castor oil resulted in the highest surface oil thickness owing to its viscosity, which correlated with diminished TENG performance (Figure S7b, Supporting Information). For reference, AFM topographic images of the sheets treated with each oil (castor oil, olive oil, paraffin liquid oil, and silicone oil) are shown in Figure S9 (Supporting Information).

Moreover, various materials are used in commercial oil‐absorbing sheets: polymers (PP/PE mixed), green tea, wood pulp, paper 1 (Hanji), and paper 2 (Hanji). Figure 3d shows the output voltage of NF‐TENG for each sheet material. Notably, PP/PE‐mixed polymer generated a distinctive high average peak voltage of 97.1 V, while other materials (green tea, wood pulp, paper 1 (Hanji), and paper 2 (Hanji)) generated low average peak voltages of 30.3, 25, 23.5, and 18 V, respectively. This is because these materials have relatively small amounts of surface charge compared to the PP/PE mixed polymer, leading to weak electrostatic induction. Detailed photographs of each sheet material are included in Figure S10 (Supporting Information).

As a counterpart to the sheet, various materials for the top and bottom electrodes can be introduced into NF‐TENG. Three representative commercial electrode materials (Nickel (Ni), Copper (Cu), and Aluminum (Al)) were selected for this study. Figure 3e shows the electrical outputs measured for these electrode materials, for which NF‐TENG produced similar average peak voltages of 95, 110.8, and 113.9 V, respectively. This is attributed to the similar electron affinities of Ni, Cu, and Al (similar triboelectric series). This implies that NF‐TENG can employ various metals as electrodes.

In terms of the design parameters of NF‐TENG, the device width and length were fixed at hand‐portable sizes of 75 and 75 mm, respectively, and the device height, sheet width, and sheet length were considered the main structural variables. As shown in Figure 3f, various device heights ranging from 6 to 14 mm at 2 mm intervals were tested. NF‐TENG generated the average peak voltage of 46.3, 75.2, 97, 81.7, and 57.8 V for 6, 8, 10, 12, and 14 mm, respectively. At a device height of 6 mm, the contact area between the sheet and electrode is large; however, the contact force decreases because the motion of the sheet is limited by the small available space. By contrast, at a device height of 14 mm, the contact force is large; however, the contact area is small owing to the excessive available space. The NF‐TENG generated a maximum electrical output at a device height of 10 mm, which has both a sufficient contact area and contact force.

Because the NF‐TENG has a closed channel shape (i.e., a side wall exists), the sheet width can only vary within the fixed device width of 75 mm. Thus, as shown in Figure 3g, the electrical output of NF‐TENG was measured for sheet widths of 30, 35, 40, 45, and 50 mm, for which the average peak voltages were 48.4, 53.8, 64.6, 63.2, and 95 V. Consequently, as the sheet width increases, the NF‐TENG exhibits a larger contact area and produces a larger electrical output. If the sheet width is fixed, the sheet length can be controlled. In this study, sheet length conditions of 50, 60, 70 mm (< device length) and 80, 90 mm (> device length) were tested, for which the average peak voltage of NF‐TENG was 37.3, 56, 94.4, 73.8, and 55.2 V, respectively (Figure 3h). When the sheet was too short (50 mm), the contact area was very small, and the electrical output decreased. As the sheet length increased from 50 to 70 mm, the sheet exhibited more dynamic behavior, and the electrical output increased. At a sheet length of 70 mm, which is the same as the device length, the mechanical fluttering motion and the resulting electrical output are maximized (largest contact force). When the sheet length was greater than the device length (80–90 mm), the sheet motion became irregular and unstable; thus, the electrical output was increasingly reduced.

The wind speed is an important environmental variable for the real‐life use of NF‐TENG. In this regard, as shown in Figure 3i, a broad wind speed range of 4–15 m s^−1^ was considered. NF‐TENG generated average peak voltages of 26.7, 87.3, 101, 115.1, 124.1, and 128.4 V for wind speeds of 4, 6, 8, 10, 12, and 15 m s^−1^, respectively. With an increase in the wind speed, the fluttering behavior of the sheet became more dynamic and stronger, and the electrical output performance of NF‐TENG increased. NF‐TENG can be utilized in both daily and extreme wind‐harvesting scenarios.

### 2.4 Application Demonstrations of NF‐TENG

The electrical output performance of NF‐TENG was evaluated under various external load resistances. Thus, average peak output voltage, output current, and power were checked for load resistance from 1 to 1 GΩ given a breeze wind speed of 6 m s^−1^. As the load resistance increased from 1 to 1 GΩ, the average peak output voltage increased from 0 to 103 V, and the average peak output current decreased from 11 to 0 µA, respectively. The peak power (instantaneous power) was calculated by multiplying the average peak output voltage by the current; the maximum peak power of NF‐TENG was 468 µW at a load resistance of 300 MΩ (Figure S11, Supporting Information).

However, the peak value does not reflect the output waveform information (such as peak frequency and peak width), which varies depending on the load resistance. Therefore, the RMS output voltage, RMS output current, and average power (RMS power) were computed using Equations ((6), (7), (8)).

(6) $$\text{RMS voltage}=\sqrt{\frac{\int V^{2}\left(t\right)dt}{T}}-\sqrt{\frac{\int {V_{Noise}}^{2}\left(t\right)dt}{T}}$$

(7) $$\text{RMS current}=\sqrt{\frac{\int I^{2}\left(t\right)\mathrm{dt}}{T}}-\sqrt{\frac{\int {I_{\mathrm{Noise}}}^{2}\left(t\right)\mathrm{dt}}{T}}$$

**Figure 4:**
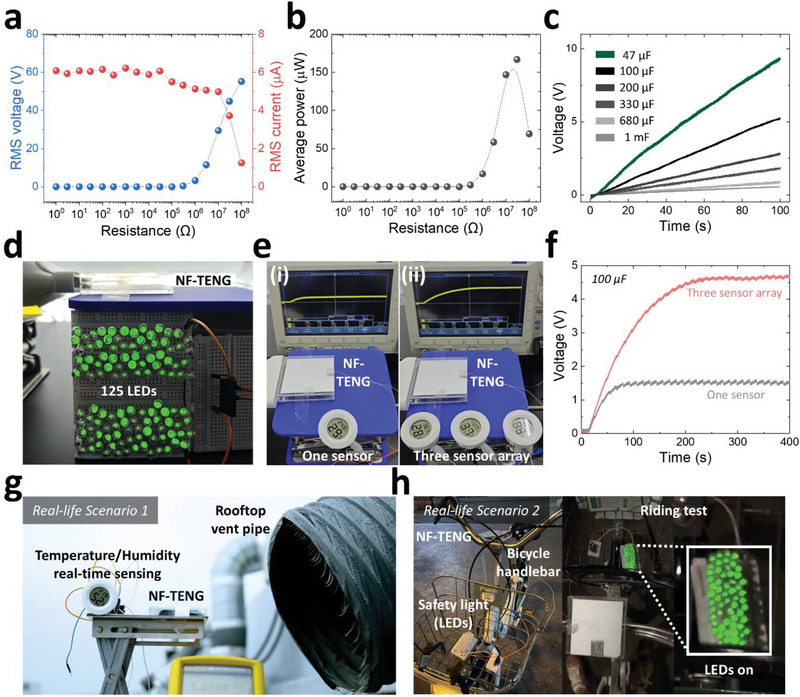
Application demonstration of NF‐TENG. a) RMS output voltage, b) current, and c) average power according to external load resistance. d) 125 LED array and e) commercial sensors driven by NF‐TENG under fixed wind input of 6 m s^−1^. f) Capacitor charging graph showing continuous operation of the commercial sensor array. g) Real‐life scenario 1: Wind harvesting at rooftop vent pipe for outdoor temperature‐humidity sensing. h) Real‐life scenario 2: wind harvesting during bicycle riding for safety light illumination.

(8) Average power=RMS voltage×RMS current

where *V(t)* and *I(t)* are the raw output voltage and current of the NF‐TENG, respectively; *V_Noise_(t)* and *I_Noise_(t)* are the raw voltage and current with respect to the ambient electric noise, respectively; and *T* is the total measurement time. With an increase of the load resistance from 1 to 100 MΩ, the RMS output voltage increased from 0 to 55 V, and the RMS output current decreased from 6.1 to 1.2 µA (**Figure** **4**a). NF‐TENG produced a maximum average power of 166 µW at a load resistance of 30 MΩ, given a breeze wind speed of 6 m/s (Figure 4b).

The electrical output generated by NF‐TENG can charge a commercial energy‐storage unit, such as a capacitor, after rectification. Figure 4c shows the charging performance of NF‐TENG for commercial electrolytic capacitors with high capacitances of 47, 100, 200, 330, 680 µF, and 1 mF, where the charging circuit only consists of NF‐TENG, a full‐wave bridge rectifier, and a capacitor. To demonstrate the peak output and charging performance at the laboratory level, 125 LED and commercial sensor driving tests were conducted, given a fixed breeze wind input of 6 m s^−1^. A single NF‐TENG brightly illuminated the 125 LED array in real time (Figure 4d). The LED illumination process and circuit used are shown in Movie S2 and Figure S12 (Supporting Information). A single NF‐TENG also operated one commercial temperature‐humidity sensor (rated voltage: 1.5 V; Figure 4e‐i) or an array of three sensors (total rated voltage: 4.5 V) (Figure 4e‐ii) in real‐time, where a 100 µF capacitor was used to continuously supply energy through repeated charging and discharging. The capacitor was rapidly charged and maintained the charging‐discharging voltage at ≈1.5 V for a single sensor and ≈4.5 V for an array of three sensors (Figure 4f). The sensor operation process and circuit are shown in Movie S3 and Figure S13 (Supporting Information).

As a final demonstration in real life, NF‐TENG was utilized in two wind‐harvesting scenarios: stationary and mobile. First, NF‐TENG was installed in front of a building's rooftop vent pipe (distance: ≈20 cm) to harvest exhaust air in a fixed state. The same charging circuit, composed of a full‐wave bridge rectifier and a capacitor, was used. A single NF‐TENG successfully charged a 100 µF capacitor to 1.5 V in 140 s, and then a commercial humidity‐temperature sensor was activated to detect outdoor environmental information in real time when the discharge switch was connected (Figure 4g; Movie S4, Supporting Information). A multimeter was used to determine the capacitor charging voltage. Next, NF‐TENG was installed on a bicycle handlebar to harvest the wind generated while moving. NF‐TENG was connected to a rectifier and 65 LEDs and then successfully lit an LED array that could function as a self‐powered safety light in a riding test (Figure 4h; Movie S5, Supporting Information).

Moreover, humidity is an important factor in TENG output performance. In Figure S14 (Supporting Information), the output voltages of the oil‐absorbing sheet with and without silicone oil are compared. The humidity experiment was conducted as follows: 1) TENG and the operating humidifier were placed in a closed chamber. The TENG did not operate until the RH (relative humidity) reached 85% (before 0 s). 2) When RH reached 85%, TENG operation started, and the humid air in the chamber was immediately exhausted (after 0 s). As a result, for 650 s, the oil‐absorbing sheet without oil showed unstable and slow output recovery owing to humidity and sheet damage. In contrast, the oil‐absorbing sheet with oil (this study) exhibited relatively stable and fast recovery because the nano‐oil barrier is resistant to humidity, causes less mechanical damage, and smooths the fluttering motion.

Scalability is also important for practical implementation. Thus, the electrical output and charging performance of NF‐TENG for the different sheet sizes (1 cm × 3 cm, 2 cm × 4 cm, 3 cm × 5 cm, 4 cm × 6 cm, and 5 cm × 7 cm) were checked; 5 cm × 7 cm is the maximum size of the commercial oil‐absorbing sheet used in this work. Here, the overall device size ratio of NF‐TENG itself was matched according to each sheet size. As a result, as the sheet size increased from 1 cm × 3 cm to 5 cm × 7 cm, the electric output and charging performance of NG‐TENG also increased because the effective contact area increased. In the charging test, the 47 µF capacitor was used and measured for 100 s (Figure S15, Supporting Information). Furthermore, considering the limitation of the maximum sheet size, a scale‐up experiment by laterally stacking two NF‐TENG was conducted. When the same wind speed (6 m s^−1^) was applied, the outputs of single NF‐TENG and double‐stacked NF‐TENG were the same; however, the charging performance of double‐stacked NF‐TENG was significantly improved as the number of peaks increased after the output was rectified. For the 47 µF capacitor, single NF‐TENG charged 9.5 V for 100 s, and double‐stacked NF‐TENG charged 16.4 V for 100 s (Figure S16, Supporting Information). As more NF‐TENGs are stacked, the charging speed is expected to increase proportionally. In conclusion, NF‐TENG can be scaled down or up to fit various real‐world application situations, and its performance can be adjusted according to the scale. In addition, from an engineering perspective, a single NF‐TENG has a low fabrication cost because it is composed of inexpensive sheets, electrodes, oil, and substrate materials, and if the oil infusion step presented in this work is automated in the future, it is expected that simple mass production will be realized.

## 3 Conclusion

In summary, a wind‐driven nano‐oil‐barrier‐based fluttering triboelectric nanogenerator (NF‐TENG) was proposed. The nano‐oil barrier was fabricated through simple oil‐infusion steps and was maintained even under contact motion, providing outstanding long‐term mechanical stability (lubrication effect) and electrical stability (no output reduction). The nano‐oil barrier was carefully investigated based on AFM imaging and electrical‐mechanical measurements. Compared to other control groups, only NF‐TENG maintained 95% output performance (from 100% initial output) even after 970 000 cycles, and its original sheet shape was not damaged. The electricity‐generation mechanism of NF‐TENG was established according to the high‐speed mechanical fluttering behavior of the sheet. The design variables were thoroughly analyzed and optimized based on the average peak voltage values (oil material: silicone oil, olive oil, and paraffin liquid oil; sheet material: PP/PE mixed polymer; electrode material: Al, Cu and Ni; Device height: 10 mm; sheet width: 50 mm; and sheet length: 70 mm). The average peak voltage of the optimized NF‐TENG increased from 26.7 to 128.4 V with an increase in wind speed (4 to 15 m s^−1^). The optimized NF‐TENG generated a peak power of 468 µW (peak frequency: 100 Hz) at the load resistance of 300 MΩ and an average power (RMS power) of 166 µW at the load resistance of 30 MΩ, given a breeze wind speed of 6 m s^−1^. NF‐TENG demonstrated its functionality in two real‐life scenarios: 1) A single NF‐TENG harvested air flow from a roof vent pipe, charged a 100 µF capacitor, and operated a commercial temperature/humidity sensor. 2) A single NF‐TENG was installed on a bicycle handlebar, harvested the wind generated during bicycle riding, and illuminated an LED array that could be used as a bicycle safety light. In this study, a commercial oil‐absorbing sheet with an internally porous structure was used; however, in a follow‐up study, new oil‐absorbing sheets will be fabricated, controlling porosity.

## 4 Experimental Section

### Fabrication of NF‐TENG

The optimized NF‐TENG was fabricated as follows: First, the top and bottom acrylic plates (thickness: 2 mm) were cut to 75 mm in width and 75 mm in length and were supported perpendicularly with other acrylic side plates. The side plates were fabricated with dimensions of 1 cm height and 75 mm length, featuring small inlets designed to hold the flagpole with a height of 2 mm and a depth of 3 mm. Side plates were placed at the ends of the top and bottom plates to provide room for the sheets. Al electrodes (DUKSUNG Hitachi Co., South Korea) were attached to the inner surfaces of the top and bottom plates. Commercial PP/PE‐mixed oil‐absorbing sheets (Mandom Co., Japan) were cut to 7 cm in length and 5 cm in width. Subsequently, commercial silicone oil (Shin‐Etsu Chemical Co., Japan) was applied to the sheet using the proposed oil‐infusion steps. The end of the shorter line of the sheet was connected to the polymer flagpole (1 mm diameter) using tape.

### Materials

Commercial PP/PE‐mixed oil‐absorbing sheets (Mandom Co., Japan), green tea (GOWOONSESANG COSMETICS Co., South Korea), wood pulp (OLIVE YOUNG Co., South Korea), Paper 1 (Hanji; OLIVE YOUNG Co., South Korea), and Paper 2 (Hanji; Parisindo Pratama Co., Indonesia) were used. Commercial oils were used: silicone oil (Shin‐Etsu Chemical Co., Japan), olive oil (DAEJUNG CHEMICALS & METALS Co., South Korea), paraffin liquid (DAEJUNG CHEMICALS & METALS Co., South Korea), and castor oil (Sanchun Chemical Co., South Korea). A universal absorbent pad (clean paper, KM Co., South Korea) was used during the proposed oil infusion steps.

### Electrical Measurements

The output voltage was measured using a high‐voltage differential probe (THDP0200, Tektronix Co.) and a mixed‐domain oscilloscope (MDO 34, Tektronix Co., USA). The output current was measured using a low‐noise current preamplifier (SR570; Stanford Research Systems Co., USA). A commercial hair dryer (MS7001A, JMW Co., South Korea) was used to apply wind input in all experiments.

### Mechanical Measurements

The surface of the oil‐absorbing sheet was characterized using AFM (NX 10, Park Systems). For surface imaging, the pinpoint mode was employed with a nanoworld tip (CONTSCR). AFM images were captured at scan sizes ranging from 10 µm × 10 µm to 20 µm × 20 µm at a scan rate of 0.5 Hz.

A pin‐on‐disk tribometer (R&B Inc., RB100 MT) was used to assess the surface oil thickness under conditions that replicated the contact pressures in NF‐TENG applications. Normal forces corresponding to 287, 386, and 544 mN were applied during the tests. The rotation radius was set to 8 mm, and the rotational speed was maintained at 60 rpm. An aluminum pin was used to simulate interactions with the electrode material. Coefficients of friction (CoFs) were monitored at 10‐min intervals, with measurements repeated three times to ensure reproducibility. Viscosities of the oils were determined using a rotational viscometer (AMETEK, Brookfield DV2TCP‐LV Rotational). Viscosity data were used to calculate the surface oil thickness by incorporating parameters such as CoF, applied load, and contact area derived from the Hertz contact model. The Young's modulus of the sheet was measured using a material‐testing machine (Z0.5, Zwick Roell).

The surface oil thickness was assessed again via FD curve analysis using a MikroMasch tip (HQ:XSC11/Al BS). The manufacturer‐supplied tip specifications indicated a force constant of 2.7 N m^−1^. The FD curve tests were conducted with three replicates at different regions of the sample at a z‐scanner speed of 0.2 µm s^−1^. The oil thickness was calculated based on the cantilever deflection and z‐distance variations observed during the snap‐in events.

## Conflict of Interest

The authors declare no conflict of interest.

## Supporting information



Supporting Information

Supplemental Movie 1

Supplemental Movie 2

Supplemental Movie 3

Supplemental Movie 4

Supplemental Movie 5

## Data Availability

The data that support the findings of this study are available from the corresponding author upon reasonable request.
