# Supplementary material for: Nano‐Oil‐Barrier‐Based Fluttering Triboelectric Nanogenerator
Source: Adv Sci (Weinh). 2025 May 20;12(30):e02278. doi: 10.1002/advs.202502278 (PMC12376523; doi:10.1002/advs.202502278)
Supplement: Supplementary file 1 — Supporting Information [file ADVS-12-e02278-s005.docx]

Supplementary Material

Nano-oil-barrier-based fluttering triboelectric nanogenerator

*Deokjae Heo^1,†^, Jiwoong Hur^2,†^, Hyeonho Cho^2,†^, Kyunghwan Cha^2^, Jaeung Choi^2^, Moonhyun Choi^1^, Jinkee Hong^3,*^, Sunghan Kim^2,*^, Sangmin Lee^2,*^*

^1^ Center for Systems Biology, Massachusetts General Hospital, Harvard Medical School, Boston, MA 02114, USA

^2^ School of Mechanical Engineering, Chung-Ang University, 84, Heukseok-ro, Dongjak-gu, Seoul 06974, Republic of Korea

^3^ Department of Chemical & Biomolecular Engineering, College of Engineering, Yonsei University, 50 Yonsei-ro, Seodaemun-gu, Seoul 03722, South Korea

^†^ These authors contributed equally to this work.

*Corresponding authors

*E-mail: Jinkee Hong (jinkee.hong@yonsei.ac.kr), Sunghan Kim (sunghankim@cau.ac.kr), Sangmin Lee (slee98@cau.ac.kr)

**This file includes:**

Figures S1 to S16

Table S1

Movies S1 to S5


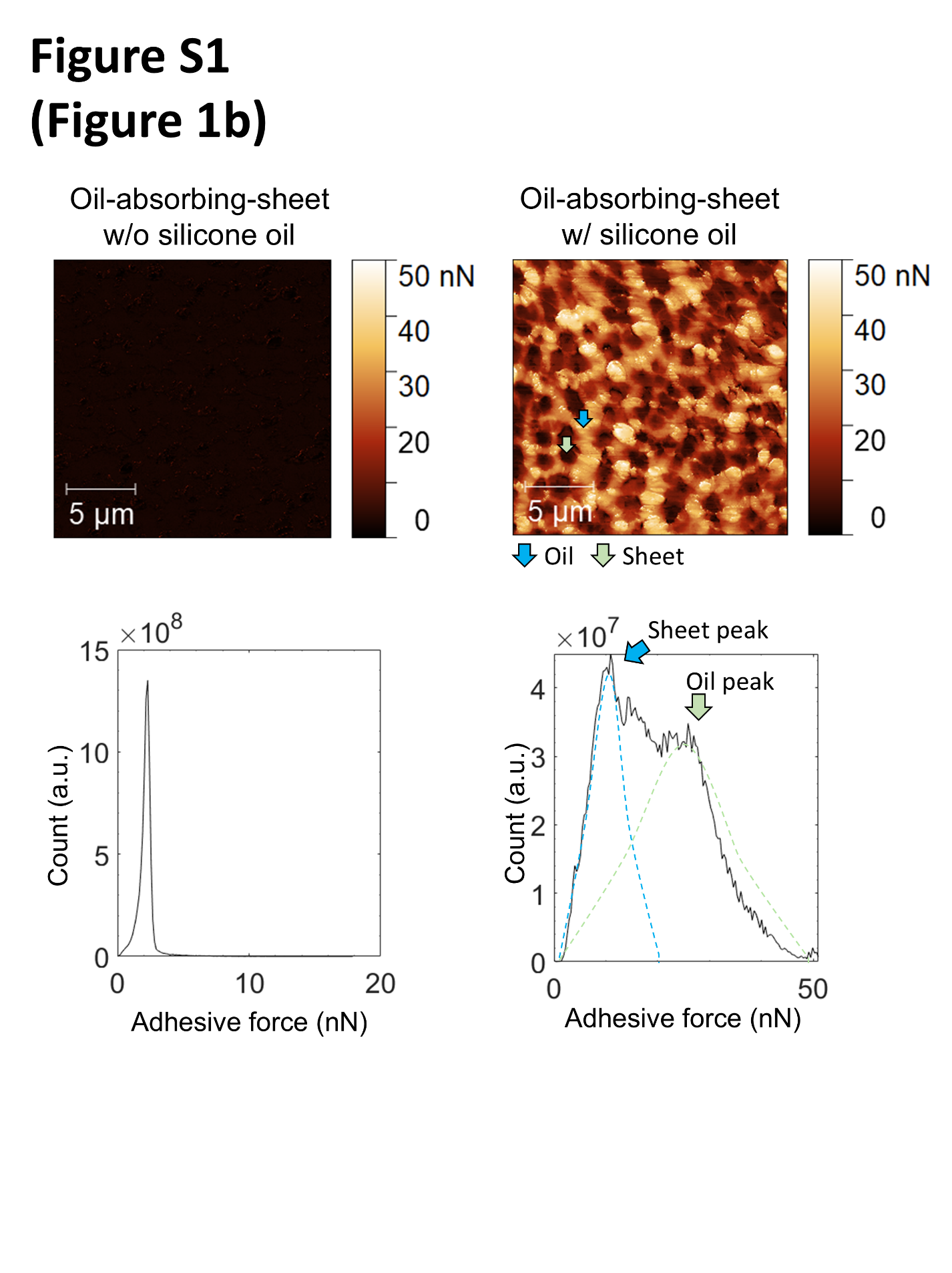


**Figure S1.** The adhesive force distribution images and corresponding graphs based on the AFM (oil-absorbing-sheet with and without silicone oil).


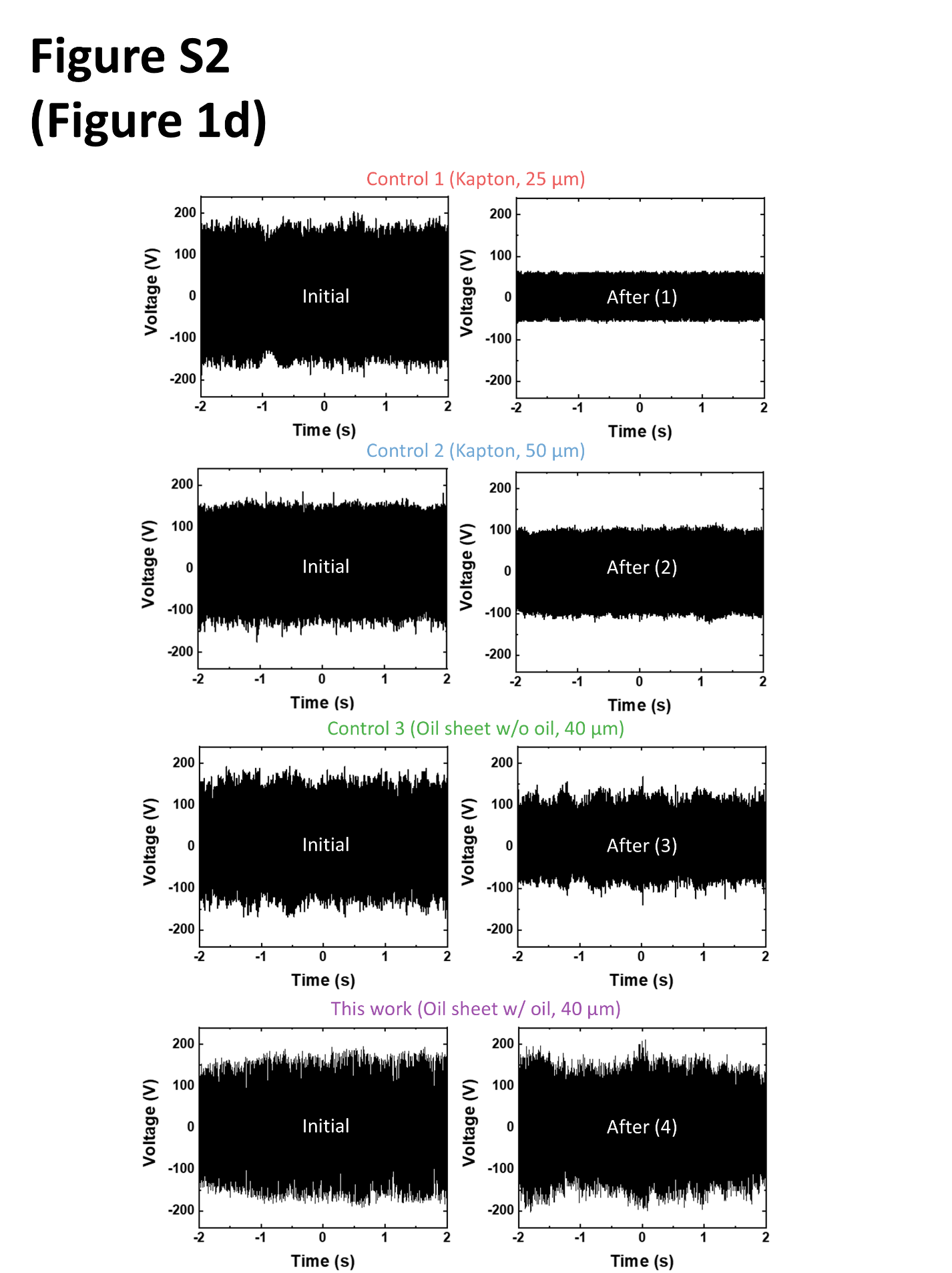


**Figure S2.** The raw output voltage graphs for long-term working cycles between this work and the three control groups.


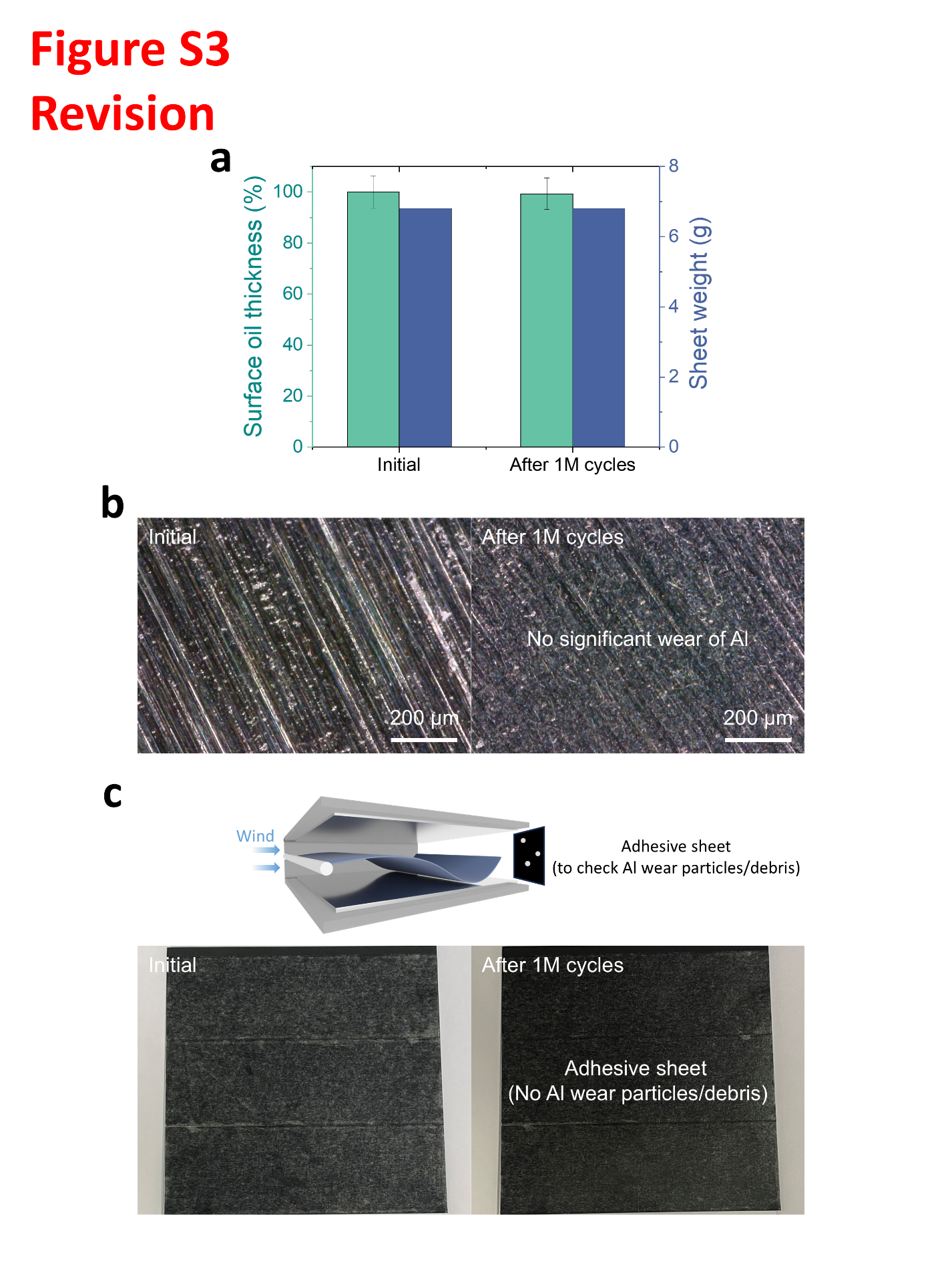


**Figure S3.** (a) oil thickness and oil-absorbing sheet weight for long-term cycles (1M cycles) to check oil leakage or sheet loss. (b) optical microscope image of the Al electrode surface for long-term cycles (1M cycles) to check the Al electrode wear. (c) Adhesive sheet observation test to check the Al electrode wear particles/debris.

| **Paper** | **TENG** | **Lubricant thickness** | **Thickness analysis** | **Test operating cycle** | **Output reduction for the test cycle** |
| --- | --- | --- | --- | --- | --- |
| This work | Wind-driven nano-oil-barrier-based fluttering triboelectric nanogenerator (NF-TENG) | 50–500 nm (Silicone oil, olive oil, paraffin liquid) | AFM (atomic force microscope) | 1,000,000 | 100 🡪 95 % |
| Zhou, Linglin, et al. Advanced Energy Materials 10.45 (2020): 2002920. | Freestanding Triboelectric Nanogenerator  (FS-TENG)  - Sliding mode  - Rotary Sliding mode | n/a  (Squalane liquid lubricant) | n/a | 500,000 (Sliding FS-TENG)  100,000 (Rotary Sliding FS-TENG) | 100 🡪 86 % (Sliding FS-TENG)  100 🡪 90 % (Rotary Sliding FS-TENG) |
| Chen, Ai, et al. Advanced Functional Materials 34.45 (2024): 2405698. | Bioinspired Self-lubricating Triboelectric Nanogenerator  (BS-TENG) | n/a  (Silicone oil) | n/a | 100,000 | 100 🡪 92 % |
| He, Wencong, et al. Research (2022). | Liquid Lubrication Promoted Triboelectric Nanogenerator  (LP-TENG) | n/a  (Silicone oil) | n/a | 500,000 | 100 🡪 90 % |
| Zhao, Jun, et al. Nano-Micro Letters 14.1 (2022): 160. | Fluorocarbon-modified Oil–Solid Triboelectric Nanogenerator  (FO-TENG) | n/a  (Paraffin oil) | n/a | 30,000 | 100 🡪 90 % |
| Zhao, Zirui, et al. Tribology International 191 (2024): 109163 | Rotary Freestanding Triboelectric Nanogenerator  (Grease-lubricated RF-TENG) | n/a  (PTFE grease, silicone grease) | n/a | 180,000 | 100 🡪 80 % |

**Table S1**. Quantitative comparison between existing lubrication TENGs^[1]^ and NF-TENG (this work)


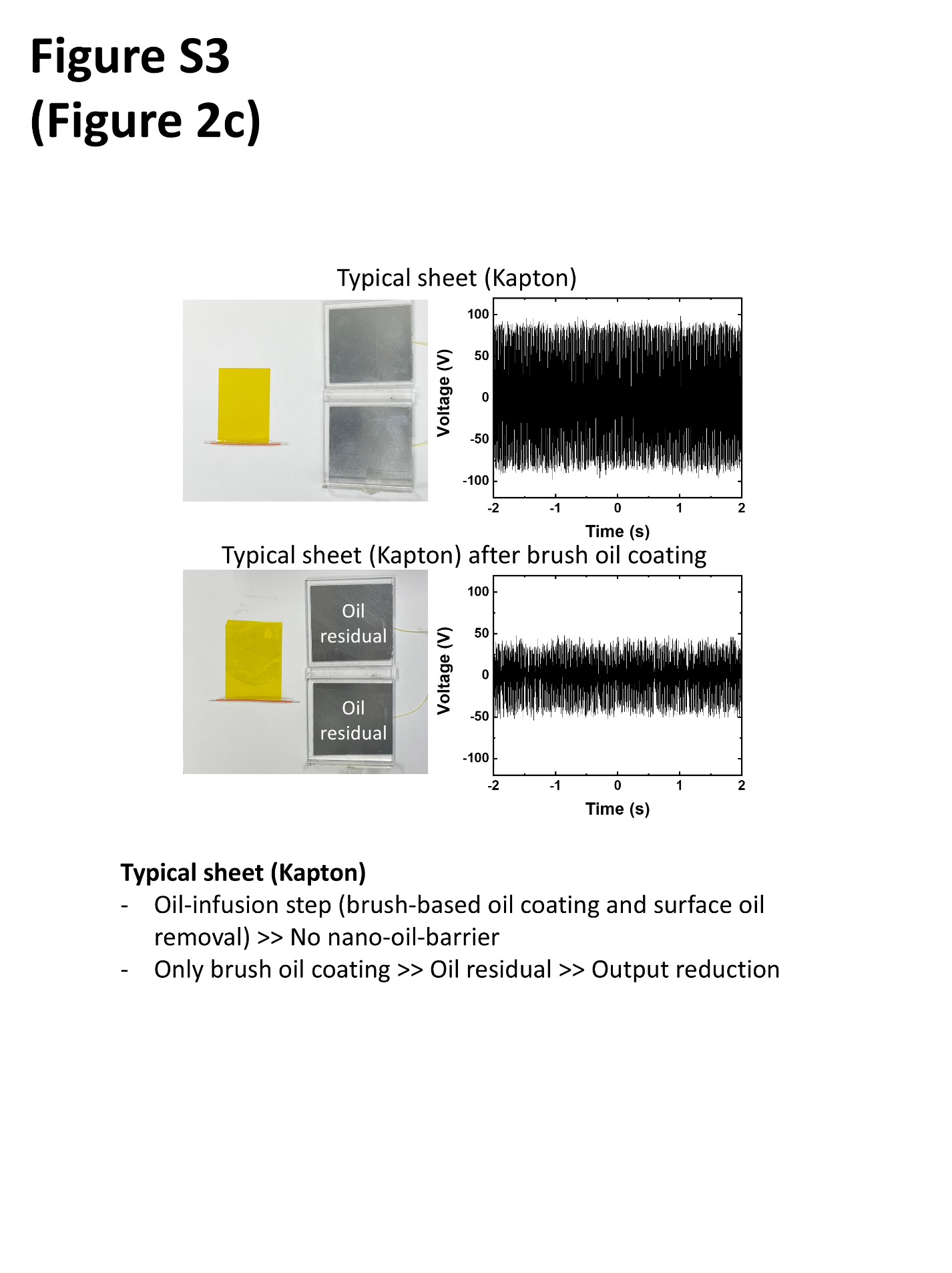


**Figure S4.** The electrical output of typical nonporous sheet (Kapton) before and after brush oil coating.


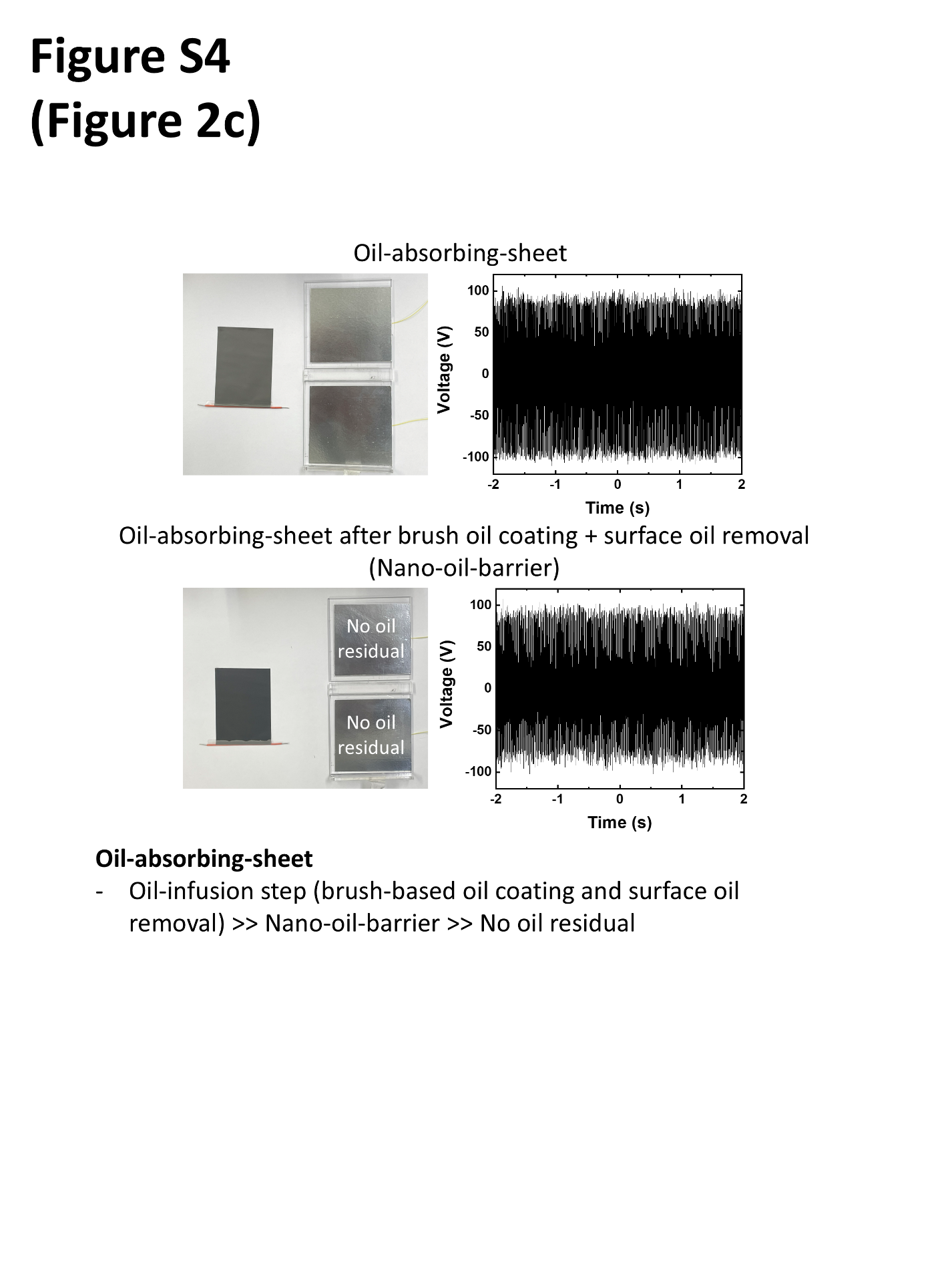


**Figure S5.** The electrical output of oil-absorbing-sheet before and after oil-infusion steps (brush oil coating/surface oil removal).


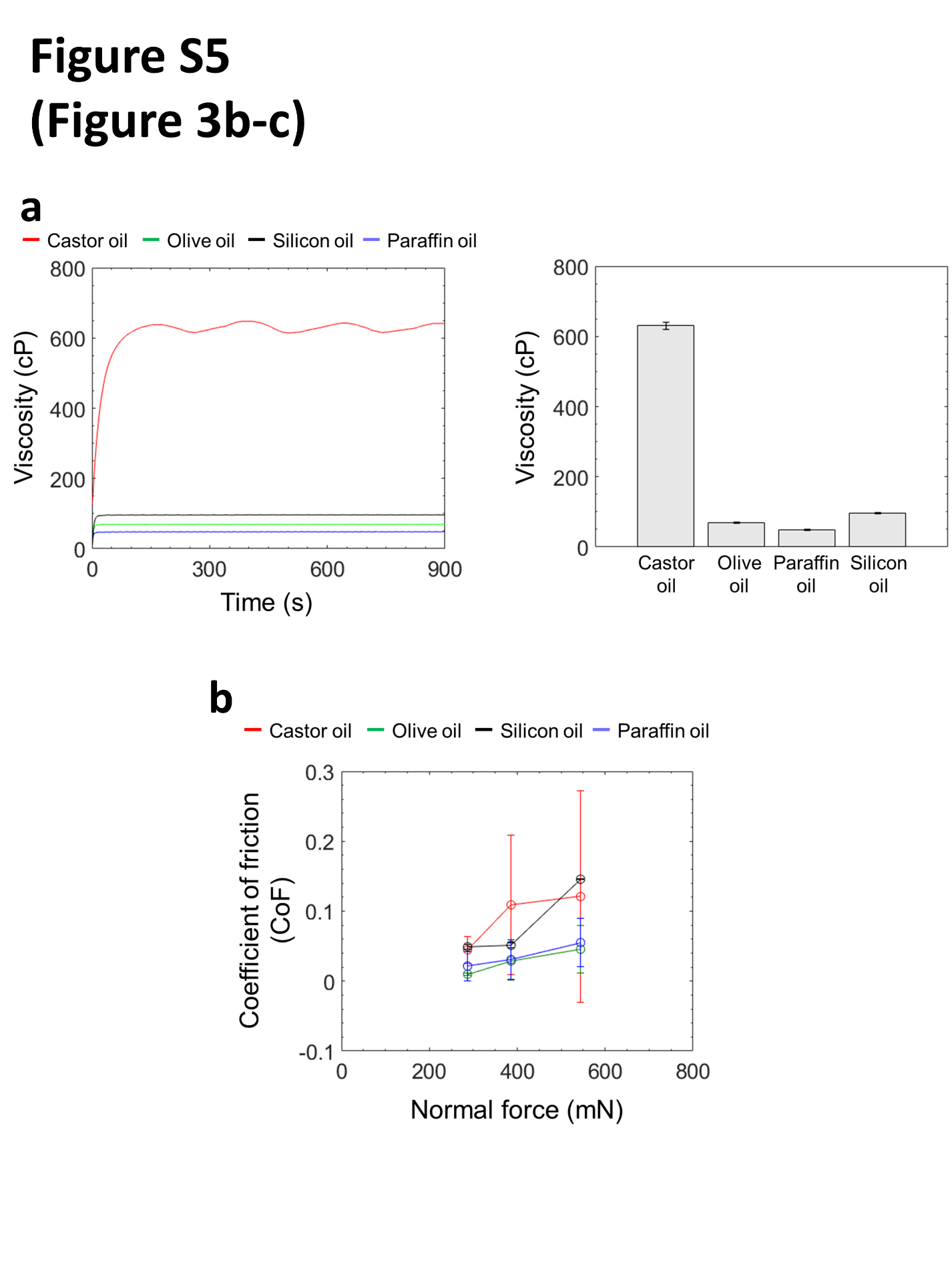


**Figure S6.** (a) The viscosity measurements for castor oil, olive oil, silicone oil, and paraffin liquid oil. (b) The coefficient of friction for castor oil, olive oil, silicone oil, and paraffin liquid oil determined under various normal forces.


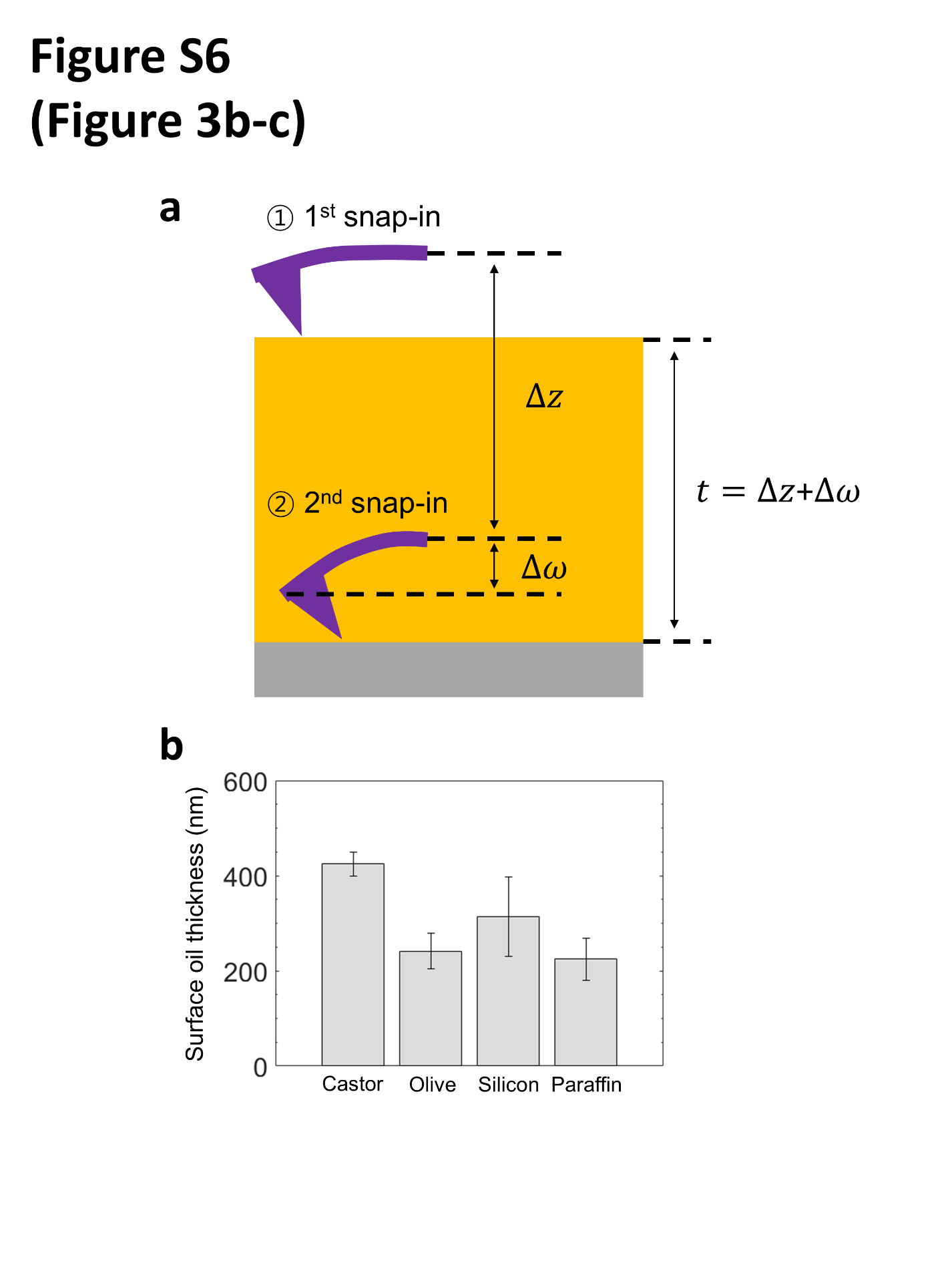


**Figure S7.** (a) The oil thickness measurement using the FD curve test. (b) The obtained oil thickness of castor oil, olive oil, silicone oil, and paraffin liquid oil.


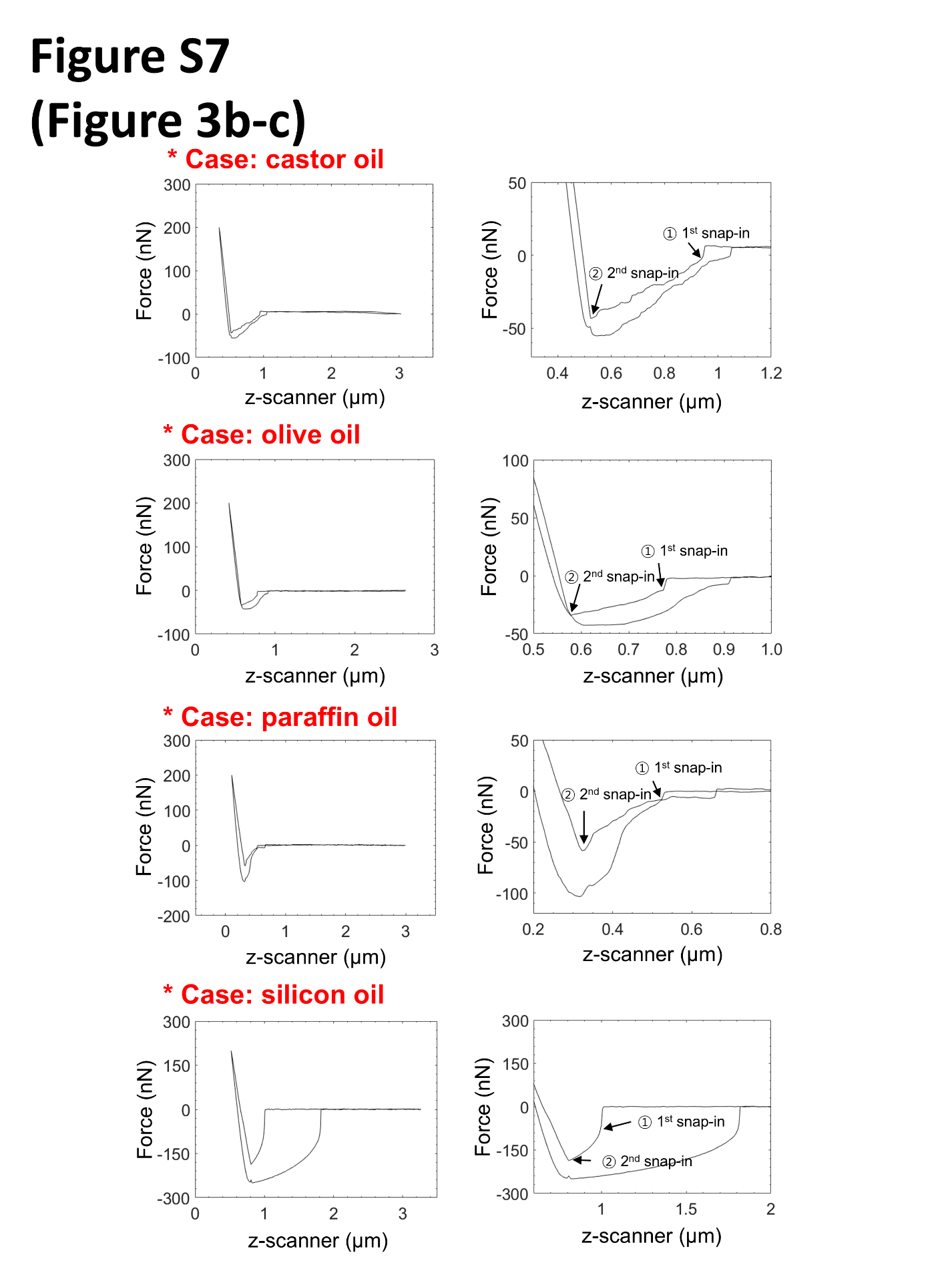


**Figure S8.** The snap-in phenomenon observed in the FD curve test for castor oil, olive oil, paraffin liquid oil, and silicone oil.


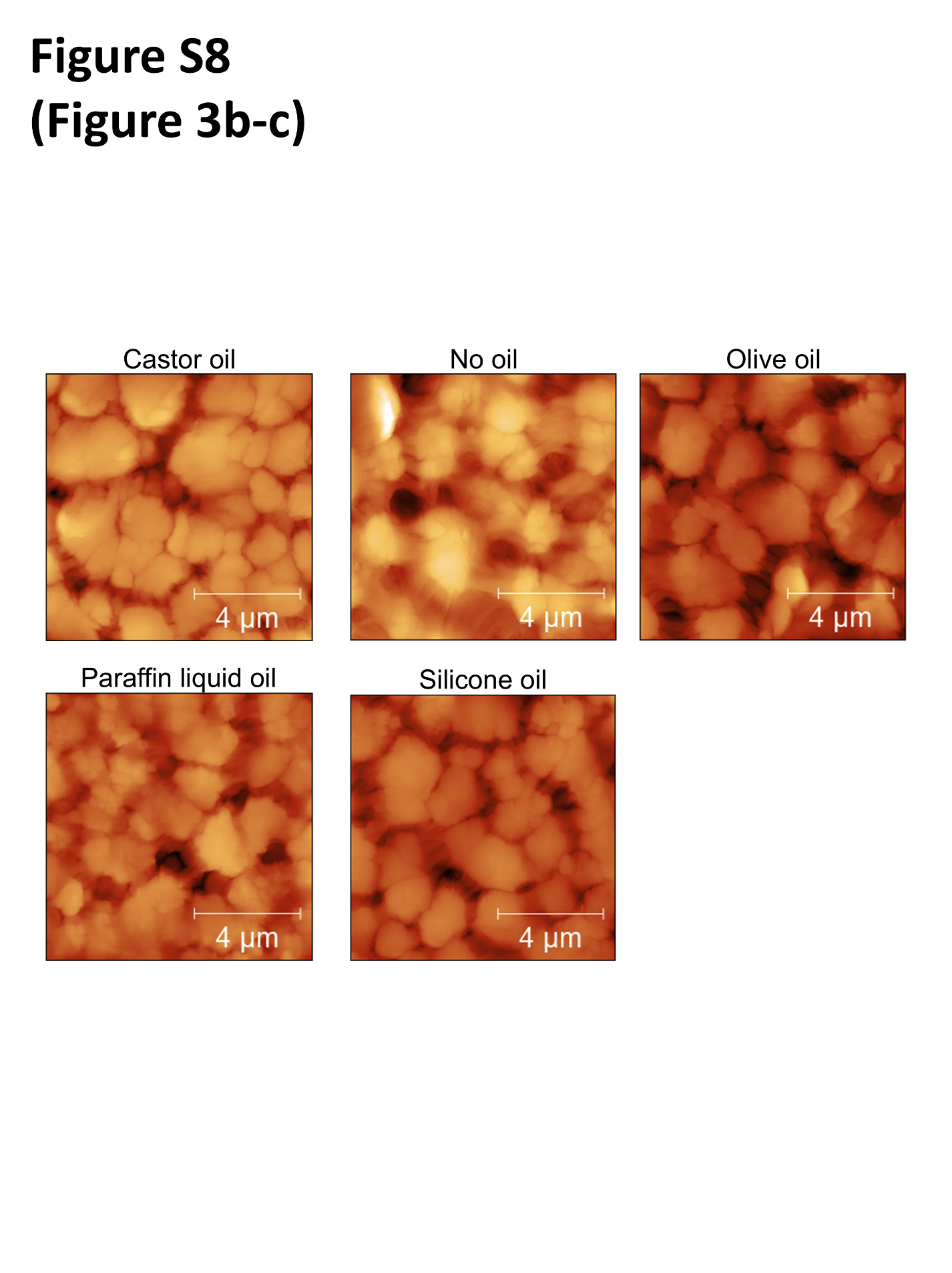


**Figure S9.** The surface topography (z-scale: 1.5 μm) of sheets that absorb castor oil, olive oil, paraffin liquid oil, and silicone oil, as well as sheets that do not absorb oil.


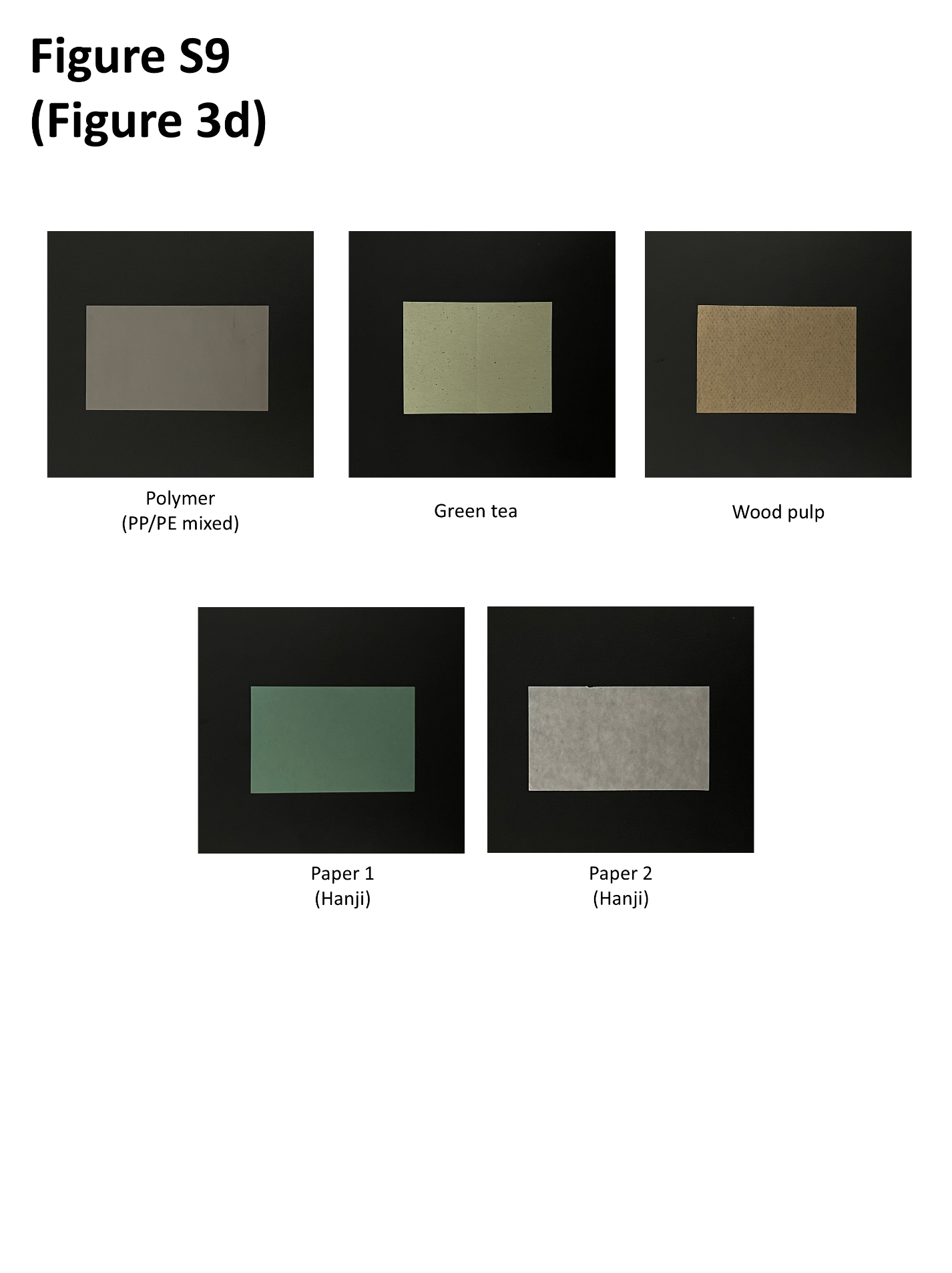


**Figure S10.** The photographs of each sheet material used for commercial oil-absorbing-sheet (Polymer (PP/PE mixed), Grean tea, Wood pulp, Paper 1 (Hanji) and Paper 2 (Hanji)).


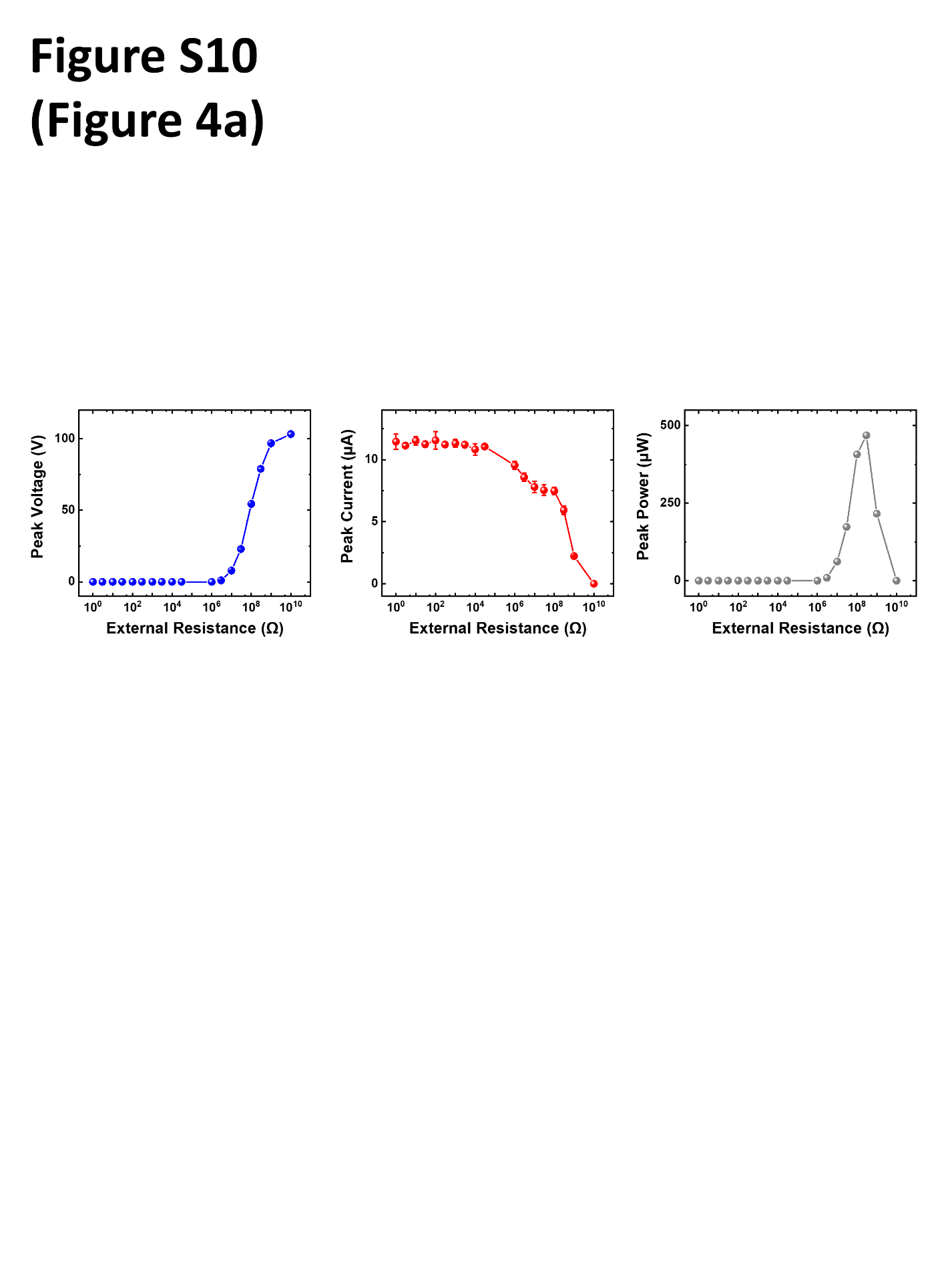


**Figure S11.** The peak output voltage, current and power according to external load resistance.


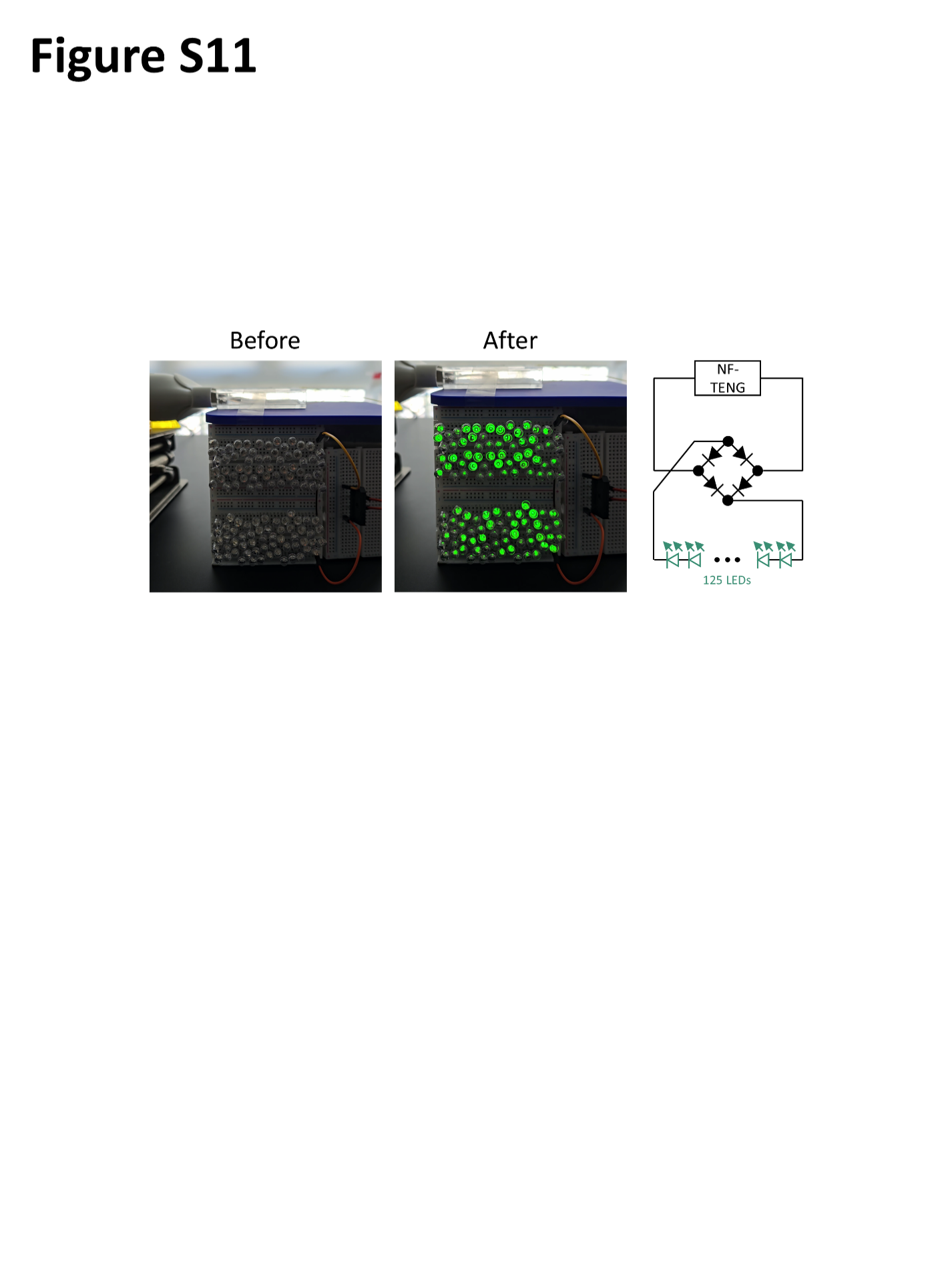


**Figure S12.** The 125 LED array illuminated by single NF-TENG and the circuit used.


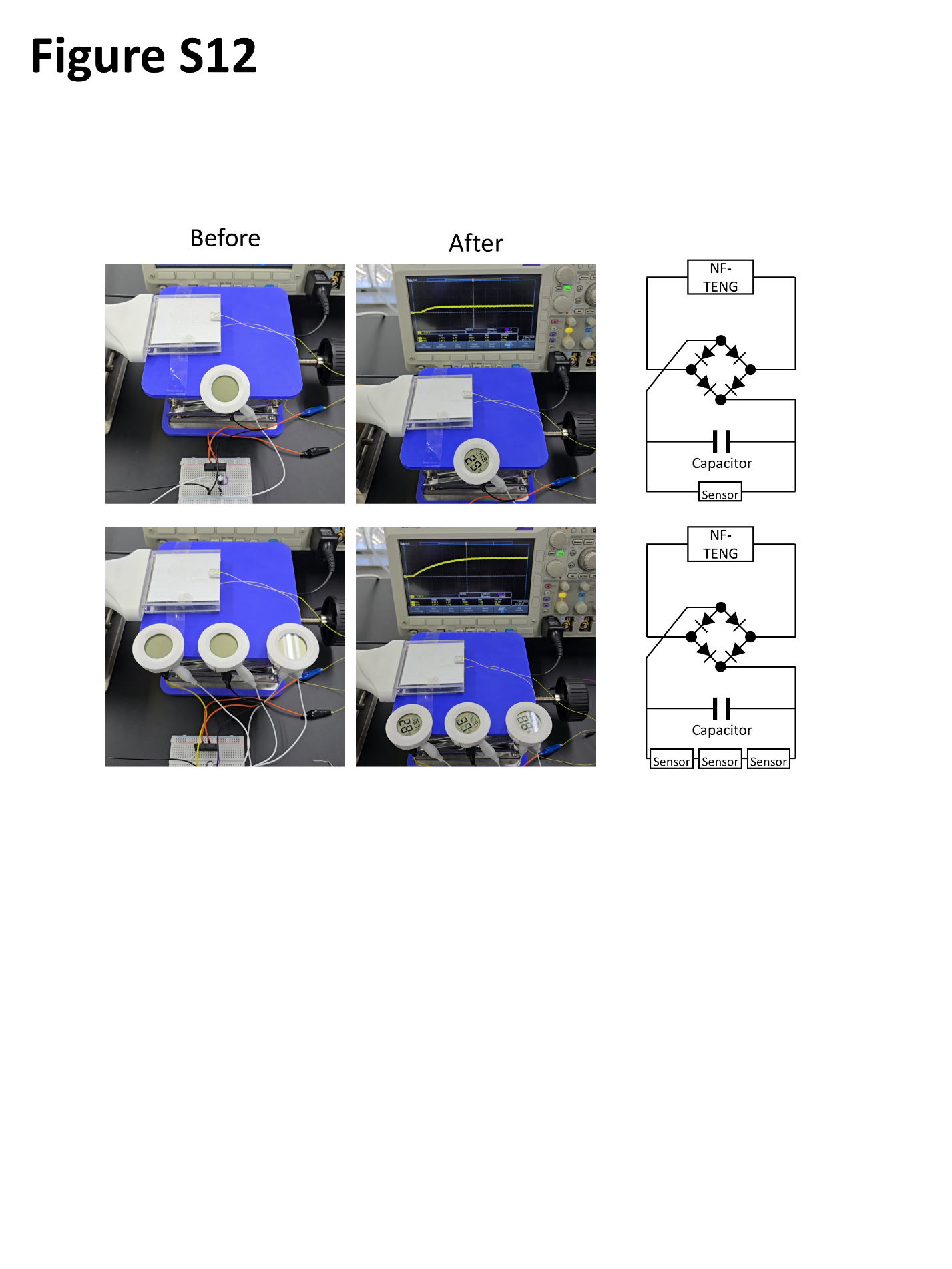


**Figure S13.** The one commercial temperature-humidity sensor and array of the three-sensor operated by single NF-TENG and the circuit used.


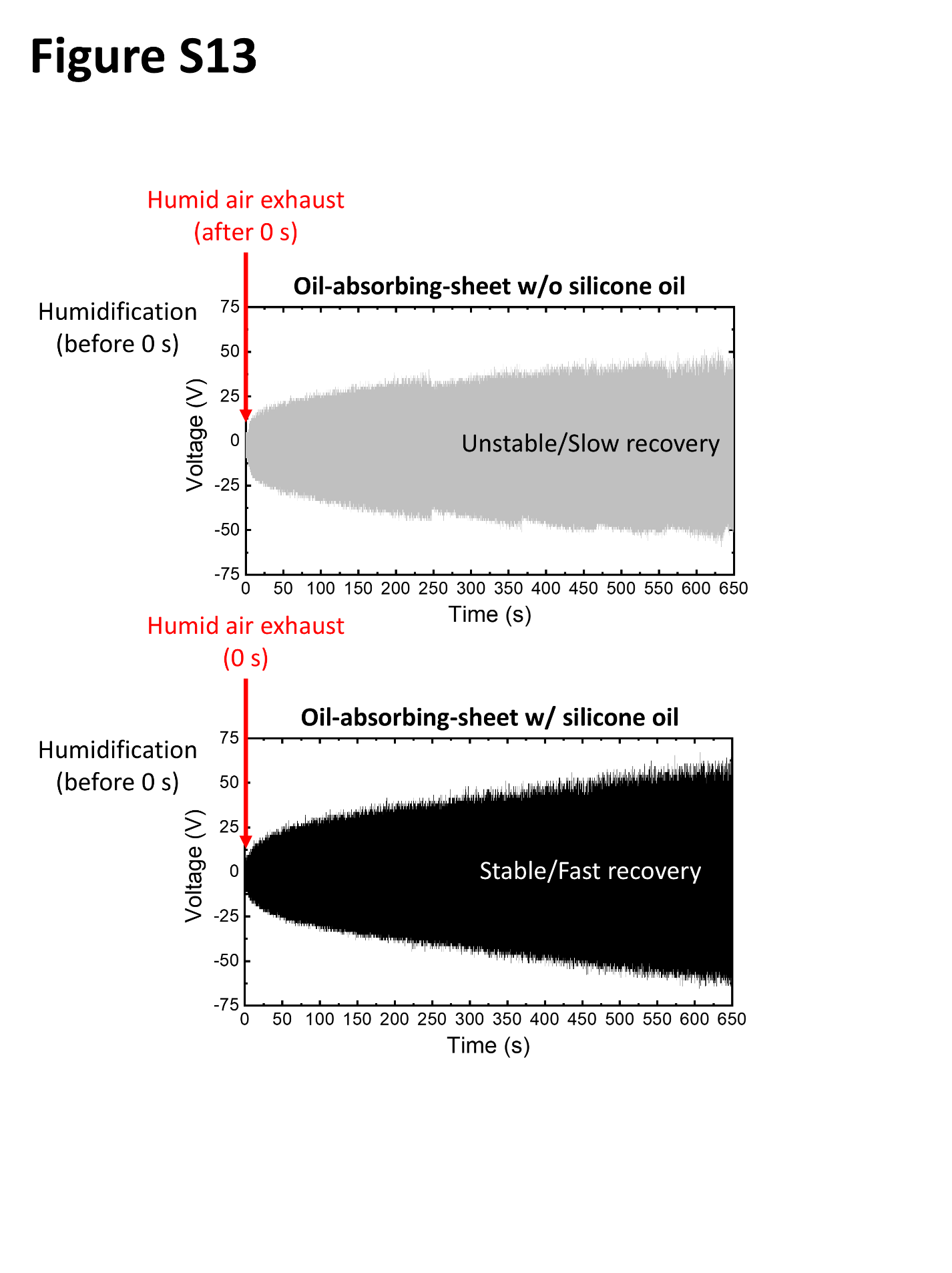


**Figure S14.** The electrical output recovery after humidity test (oil-absorbing-sheet with and without silicone oil).


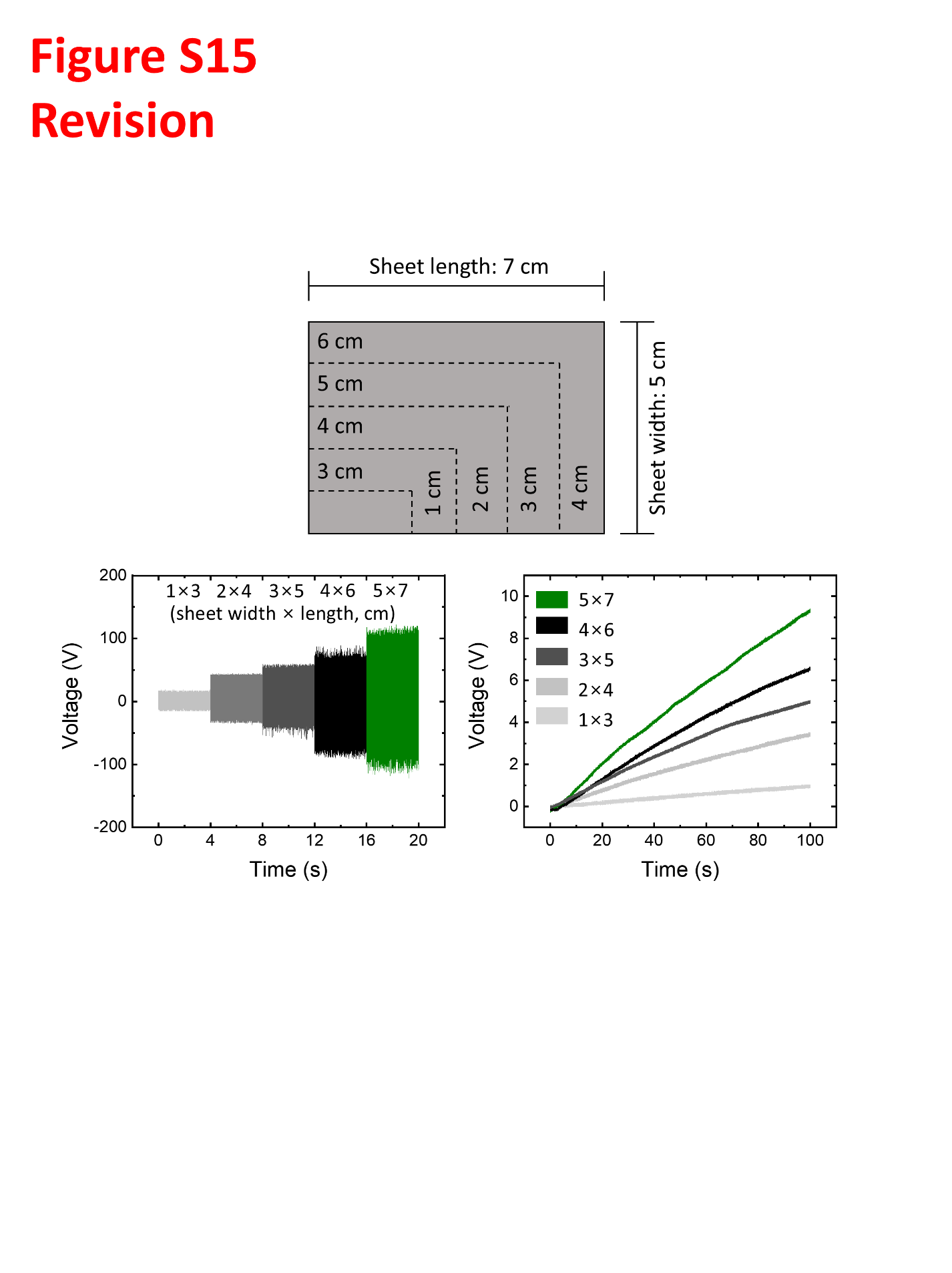


**Figure S15.** The electrical output and charging performance of NF-TENG for the different sheet sizes (1 cm × 3 cm, 2 cm × 4 cm, 3 cm × 5 cm, 4 cm × 6 cm, and 5 cm × 7 cm).


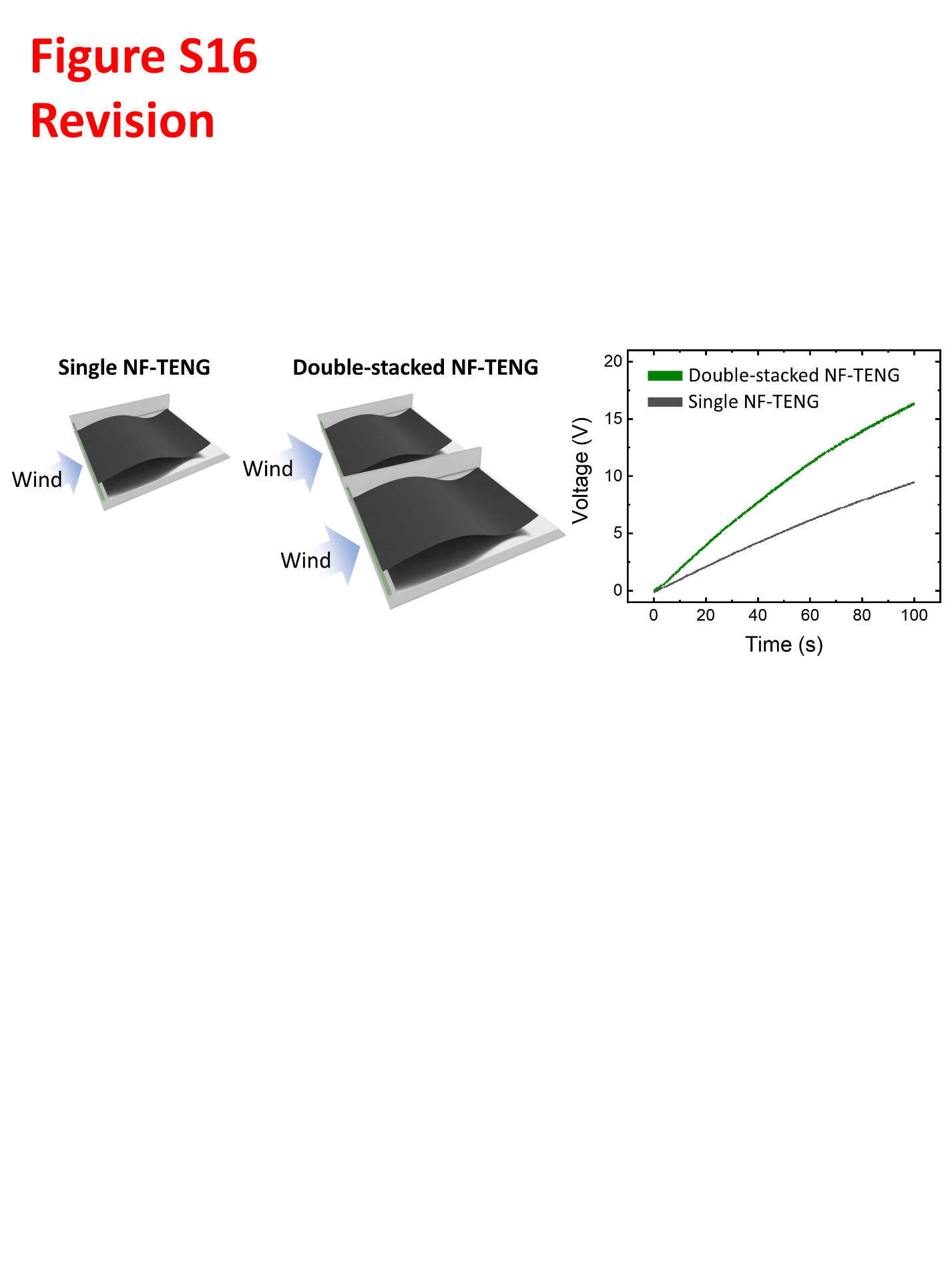


**Figure S16.** The performance comparison between single NF-TENG and double-stacked NF-TENG.

**Movie S1.** The high-speed sheet fluttering motion of NF-TENG

**Movie S2.** The LED array illuminated by NF-TENG

**Movie S3.** The single and array of commercial temperature/humidity sensor operated by NF-TENG

**Movie S4.** The LED array (illuminated by NF-TENG in real-life scenario (safety light during bicycle riding)

**Movie S5.** The single commercial temperature/humidity sensor operated by NF-TENG in real-life scenario (rooftop vent pipe)

**References (Supplementary Material)**

[1] a)A. Chen, Q. Zeng, L. Tan, T. Wang, F. Xu, J. Wang, X. Tao, Y. Yang, X. Wang, *Advanced Functional Materials* **2024**, 34, 2405698; b)W. He, W. Liu, S. Fu, H. Wu, C. Shan, Z. Wang, Y. Xi, X. Wang, H. Guo, H. Liu, *Research* **2022**; c)J. Zhao, D. Wang, F. Zhang, J. Pan, P. Claesson, R. Larsson, Y. Shi, *Nano-Micro Letters* **2022**, 14, 160; d)Z. Zhao, X. Wang, Y. Hu, Z. Li, L. Li, G. Ye, *Tribology International* **2024**, 191, 109163; e)L. Zhou, D. Liu, Z. Zhao, S. Li, Y. Liu, L. Liu, Y. Gao, Z. L. Wang, J. Wang, *Advanced Energy Materials* **2020**, 10, 2002920.
